# Nanoscale Prognosis of Colorectal Cancer Metastasis from AFM Image Processing of Histological Sections

**DOI:** 10.3390/cancers15041220

**Published:** 2023-02-14

**Authors:** Vassilios Gavriil, Angelo Ferraro, Alkiviadis-Constantinos Cefalas, Zoe Kollia, Francesco Pepe, Umberto Malapelle, Caterina De Luca, Giancarlo Troncone, Evangelia Sarantopoulou

**Affiliations:** 1 National Hellenic Research Foundation, Theoretical and Physical Chemistry Institute, 48 Vassileos Constantinou Avenue, 11635 Athens, Greece; 2 Dipartimento di Sanità Pubblica, Università Degli Studi di Napoli “Federico II”, via Pansini 5, 801301 Napoli, Italy

**Keywords:** nanoscale metastatic prognosis, colorectal cancer tissue, AFM imaging, variogram hierarchy

## Abstract

**Simple Summary:**

This work advances colorectal cancer (CRC) metastatic prognosis by identifying morphological metastatic markers from image processing of atomic force microscopy (AFM) images of CRC histological sections. High orders of variograms of residuals of Gaussian-filtered images define metastatic/non-metastatic thresholds with 97.7 nm spatial resolution. The metastatic/non-metastatic differentiation defines irreversible hierarchical and complexity levels.

**Abstract:**

Early ascertainment of metastatic tumour phases is crucial to improve cancer survival, formulate an accurate prognostic report of disease advancement, and, most importantly, quantify the metastatic progression and malignancy state of primary cancer cells with a universal numerical indexing system. This work proposes an early improvement to metastatic cancer detection with 97.7 nm spatial resolution by indexing the metastatic cancer phases from the analysis of atomic force microscopy images of human colorectal cancer histological sections. The procedure applies variograms of residuals of Gaussian filtering and theta statistics of colorectal cancer tissue image settings. This methodology elucidates the early metastatic progression at the nanoscale level by setting metastatic indexes and critical thresholds based on relatively large histological sections and categorising the malignancy state of a few suspicious cells not identified with optical image analysis. In addition, we sought to detect early tiny morphological differentiations indicating potential cell transition from epithelial cell phenotypes of low metastatic potential to those of high metastatic potential. This metastatic differentiation, which is also identified in higher moments of variograms, sets different hierarchical levels for metastatic progression dynamics.

## 1. Introduction

An estimated 19.3 million new cancer cases and almost 10.0 million cancer deaths occurred in 2020 worldwide [1], and metastasis is the leading cause of mortality. Tumour metastasis is the migration of cancer cells from the primary tumour cores to the lymph nodes, tissues, or distant organs. Metastasis is responsible for 90% of colorectal cancer (CRC) deaths; therefore, early diagnosis is critical for patient survival. Metastasis is a complex process that involves morphological adjustments and the attachment of cancer cells to other cells and the extracellular matrix (ECM). It represents a key hallmark [2] of malignance’s progression towards a higher pathological state. Therefore, indexing assessments of the metastatic state and its early prediction is fundamental to enlighten cancer progression, improve early cancer prognosis, and develop therapeutic schemes [3]. Tissue microenvironmental factors, including stiffness and topography (the nuclei’s shapes, morphology, and texture specificity), contribute to the targeting preferences of metastatic cancers [4,5,6,7] because biological and mechanical/topographic parameters are associated with cancer cell proliferation, migration, and metastasis [8,9]. Cancer cells regulate their stiffness to match the ECM local environment by adjusting their viability in different ECM structural proteins’ complexes and topographical environments [10].

Metastasis of variable percentages may arise in all stages, indicating that common histological and cytological findings are necessary but insufficient to identify high-risk characteristics and predict metastatic phases. Likewise, to improve patients’ survival, it is mandatory to ascertain the tumour’s stage accurately, formulate a universal prognostic report about disease progression, and, most importantly, identify the metastatic phase and heterogeneity of primary cancer cells as early as possible [11]. Even though the latest CRC-TNM classification protocols of regional lymph nodes are considered, and each pathological stage is further subdivided [12], early stage and novel classification schemes are needed; therefore, this research aims to establish arithmetic biomarkers for early and reliable tumour diagnosis and metastasis prognosis. The correlation between metastasis and tumour histological alterations was recognised in the early mid-nineteenth century. Since then, optical and electronic microscopy has been applied for routine cancer diagnostics through visual interpretation of ultra-thin, two-dimensional tissue sections, which histopathologists use to decide whether tissue regions are cancerous and to classify the malignancy level [13].

Still, diagnosis and classification of cancer are operator-dependent, and thus, they are imperilled to error. In addition, negative factors include the inherent limitations of magnification, field of view, contrast, and the small focal depth of optical systems [14]. Consequently, in addition to optical imaging, state-of-the-art histological image analysis software and texture algorithms exploiting the microscopic variations of cells’ shapes and tissue morphologies are needed for early and reliable prediction of metastasis. Along these lines, a novel methodology of probing the mechanics of tumours emerged as a supportive method to find the link between the mechanical properties of single tumour cells and their metastatic potential [15,16,17]. However, although several techniques exist, including atomic force microscopy (AFM) [4,18,19,20], to measure the mechanical properties of single cells, information on the mechanics of tumour cells in the ECM is missing because most measurements are made on cultured tumour cells [21]. Moreover, each method has a particular set of parameters that do not consider patient-to-patient variations, which is an additional drawback in comparing different studies.

Machine vision and learning methods were also applied as complementary approaches to microscopic histopathological examination and molecular-based approaches for cancer prediction and prognosis [22,23]. The established digital histopathology image analysis technique is based on tissue image classification and tiny segmented structures, including nuclei and cells [24]. However, machine learning still experiences numerous technical and organisational challenges and limitations because of the complexity of tissue morphology, tumour heterogeneity, and the diversity of shapes, locations, and sizes of tumour segmentation. In addition, developing accurate and efficient algorithms is still challenging [22].

Likewise, the mathematical modelling and dynamics of complex natural systems, including tumour advancement [25,26], aim to characterise architecture and decode spatial and temporal complexity and heterogeneity commonly appearing in nature [27]. Fractality, complexity, and structure statistics discriminate tags suitably from Euclidean morphometric measurements (e.g., length, volume, and density) [28], and several methods were developed to study physical entities in many different contexts [29]. For example, the generalised method of moments (GMM) is viewed as an extension of *z*-height correlation functions. Variograms are also used extensively in geology and medicine [30] to quantify images’ spatial variability and correlation distances. A variogram expresses the expected square difference between two data values separated by a distance-vector, e.g., grayscale values between pixels in optical microscopy or *z*-height values in AFM images. Overall, one- or two-dimensional variograms (1D or 2D) are visual expressions of the spatial correlation of image points.

Variograms are used in diagnosis, including spatial tissue displacement of ultrasound elastography in areas surrounding needles, image-guided neurosurgery, non-subjective evaluation of chromatin in cell proliferation and apoptosis, the magnetic resonance of 3D brain structural changes, and spatial autocorrelation stiffness differences between aortic and pulmonary valve interstitial cells. In addition, variograms, among other tools, are applied to 2D malignant breast tissue images [31], anticancer treatments [32], and differentiation between melanomas and normal skin tissues [33].

Although low spatial resolution optical imaging utilises variograms [34], an early cancer prognostic tool implies tissue structural differentiation at the nanoscale level [35]. However, a reliable, label-free, non-invasive approach for identifying and quantifying nanoscale metastatic differentiation on conventional histological sections is challenging [36]. In this direction, AFM is suitable for non-destructive 3D imaging of cells and tissues with nanometric resolution [16,37]. Primarily, the AFM-based single-molecule method provides unique biomolecular-level insights with sub-nm resolution in near-native conditions into molecular properties distributions and identification of existing subpopulations [38]. So far, few AFM studies have analysed formalin-fixed and paraffin-embedded (FFPE) cancer histological tissues because of diagnostic and prognostic constraints [39,40]. In histopathology image analysis, second-order effects and lacunarity (distribution, size of gaps between cells) [41] were proposed as marking factors. The correlation between the fractal dimension of AFM images and the z-scale factor serves as a mechanical mark of human lung carcinoma [42]. Analysis of the AFM adhesion of cells [43] reveals that fractality differences are evident when premalignant cells transform into cancerous cells [44].

Variogram analysis is based on the hypothesis that images’ statistical means and variances are independent of their pixels’ locations. In addition, statistical mean and variance commonly bear comparable values for entities in similar groups, such as the different sets of metastatic and non-metastatic CRC AFM images of histological tissues. Domain size Gaussian filtering (DSGF) variograms [30] differentiate similar but different hierarchies and complexity levels, e.g., dissimilar cognitive, memory, and functionality dynamic systems [45].

Small biological features discriminate AFM images of metastatic/non-metastatic CRC tissues in this work. A significant sensitivity improvement in differentiating metastatic/non-metastatic stages in CRC cells was obtained by applying moment variograms of residuals of Gaussian filtering and theta statistics [46] in 50 μm × 50 μm AFM cancer histological images from five different patients (three metastatic and two non-metastatic). Likewise, AFM image theta statistics incorporate inclination histograms of tiny planar segments of CRC histological sections. Theta distribution skewness can also differentiate the signatures of different hierarchical groups as metastatic and non-metastatic tissues. 

Furthermore, towards establishing early quantifying markers of metastatic phases, the differentiation between metastatic and non-metastatic tissues was approached with rescaled range, surface statistics, and phase analysis in AFM imaging. The results were compared with those from variograms and theta statistics. Noticeably, the novelty and state-of-the-art of the current work are grounded on improving metastatic differentiation by higher moments of variograms.

This tactic aims to provide insight into metastatic hierarchical levels and the dynamics of metastatic evolution by diagnosing the malignant condition of suspicious cells (typically a few) not identified via optical microscopy when subtle signs appear. We sought to identify early tiny morphological changes indicating potential cell transition from an epithelial phenotype typical of cells with low metastatic potential to a mesenchymal phenotype that marks high-mobility cell features and provides quantifying universal metastatic indexes and critical thresholds.

## 2. Materials and Methods

### 2.1. Histological Tissue Preparation 

CRC human histological tissues were prepared at the University Hospital “Federico II” in Naples, Italy and labelled using anonymous numerical codes. Human tissues were handled and prepared following the Helsinki protocol (https://www.wma.net/wp-content/uploads/2016/11/DoH-Oct2008.pdf, accessed on 9 July 2008). The tissue samples were labelled (1) according to the tumour site (right colon, transverse, left colon, or rectosigmoid), (2) the pathological classification (Cancer Control UICC, 2017, T, N, or M), (3) the vascular hematic, vascular lymphatic, and perineural invasion, and (4) the surgical resection margin status. Necrosis, neoplastic cellular percentage, desmoplasia, and tumour-infiltrating lymphocytes were assessed using optical microscopy. The mucinous acellular component was categorised as absent (<1%) or present (≤50% or >50%). 

The tumour histological sections were collected on glass slides in FFPE blocks. Before AFM imaging, they were dewaxed at 60 °C. Then, they were washed for 300 s in three steps with xylene, and xylene traces were removed in three washing steps in 100% ethanol for 300 s each time. After that, slides were further washed in 95% ethanol for 300 s and distilled in water once again for 300 s. Samples were stained with hematoxylin solution according to the instructions of the manufacturer, Mayer (Sigma Aldrich Chemie GmbH, Steinheim, Germany, 1.044 grml^−1^ at 20 °C), and dried in the air for about 600 s at 20 °C. 

### 2.2. AFM Image Analysis

Eighteen fixed histological tissues (eleven metastatic and seven non-metastatic) were imaged with Innova AFM (Bruker/Veeco, Inc., Santa Barbara, CA, USA) operating in tapping mode with a phosphorus (n)-doped silicon cantilever (RTESPA, Bruker, Madison, WI, USA) with a nominal tip diameter of 8–10 nm and a nominal spring constant of 40 N/m at a 300 kHz resonance frequency.

Surface image quality was optimised by lowering the scan rate to 0.2 Hz. All images were acquired with 50 μm × 50 μm scan sizes, 512 × 512 data point resolution, and a pixel size of 97.7 nm. In addition to height, amplitude and phase images were also recorded. The AFM was installed on a vibration isolation table (minus k technology BM-10, Inglewood, CA, USA) to compensate for regular environmental vibrations and placed inside an acoustic enclosure (Ambios technologies Isochamber, Santa Cruz, CA, USA) for thermal and building vibration isolation. The AFM imaging was performed in air at a constant ambient temperature.

### 2.3. Histological Tissue Optical Analysis 

Before AFM imaging, optical microscopy was used for metastatic identification. First, the paraffin-stained CRC histological sections were placed under a transmitted light optical microscope (Primovert microscope, Carl Zeiss Co. Ltd., Oberkochen, Germany) with magnifications of 4×, 10×, 20×, and 40×. Then, the AFM probe was positioned in the identified image areas.

### 2.4. Gaussian Filtering Residuals RMS Deviation

A 3D Gaussian filter was applied to the original image for each AFM image. The Gaussian cubic filter size (kernel) was set to 31 pixels (px) with a standard deviation *σ* of 5 px in every dimension. The residuals of the Gaussian filter (a high-pass filter that represents the small-scale roughness of the surface) consist primarily of the spatial frequencies below the cut-off wavelength (*6σ +* 1 = 31 px or ~3 μm, 1 px = 97.66 nm for 512 px × 512 px image resolution), with some leakage of higher spatial frequencies. The statistical measure of the height differences for all possible point pairs of an area at a particular scale, the root mean square (RMS) deviation D(h), was determined for each lag vector h=(±v,±p) and then scaled with the lag vectors’ magnitude using the equations below:

(1) $$D\left(h\right)=\sqrt{\frac{1}{N}\overset{l-v}{\underset{i=1}{\sum }}\overset{l-p}{\underset{j=1}{\sum }}[z{\left(x_{i},y_{i})-z\left(x_{i+v},y_{j+p}\right)\right]}^{2}},\left(v,p>0\right)\;$$

(2) $$D\left(h\right)=\sqrt{\frac{1}{N}\overset{l-v}{\underset{i=1}{\sum }}\overset{l}{\underset{j=1-p}{\sum }}[z{\left(x_{i},y_{i})-z\left(x_{i+v},y_{j+p}\right)\right]}^{2}},\left(v>0,p<0\right)$$

(3) $$D\left(h\right)=\sqrt{\frac{1}{N}\overset{l}{\underset{i=1-v}{\sum }}\overset{l-p}{\underset{j=1}{\sum }}[z{\left(x_{i},y_{i})-z\left(x_{i+v},y_{j+p}\right)\right]}^{2}},\left(v<0,p>0\right)\;$$

(4) $$D\left(h\right)=\sqrt{\frac{1}{N}\overset{l}{\underset{i=1-v}{\sum }}\overset{l}{\underset{j=1-p}{\sum }}[z{\left(x_{i},y_{i})-z\left(x_{i+v},y_{j+p}\right)\right]}^{2}},\left(v,p<0\right)\;$$

where l stands for the size of the image and N is the number of sample points separated by $\left|h\right|=\sqrt{v^{2}+p^{2}}$.

The RMS deviation as a function of lag vectors in all directions is depicted in 2D or 1D plots (variograms).

One-dimensional plots describe the RMS deviation between all points spaced apart by $h=\sqrt{v^{2}+p^{2}},$ alternatively called empirical or experimental variograms/semivariograms. The empirical variograms were calculated as the average of the square differences between the values $z(x_{i},y_{i}),z\left(x_{i+v},y_{j+p}\right)$ for all pairs of locations that fall within length intervals, h (lags).

The sill value in variograms depicts zero correlation of lag vectors, visualised with variograms’ flattening off. The analysis was made for three different image resolutions, 512, 256, and 128 px per axis, and three different Gaussian filtering standard deviation values: 2.5, 5.0, and 10.0 px.

### 2.5. Moments of Gaussian Filtering Residual Variograms

Various Gaussian filtering residual variogram moments were calculated as an extension of the previous method. For q=[0.5, 1, 2, 3, 4, 5], the generalised variogram γ(h,q) was evaluated.

(5) $$\gamma \left(h,q\right)={\left\{\frac{1}{N}\overset{l}{\underset{i=1}{\sum }}\overset{l}{\underset{j=1}{\sum }}[z{\left(x_{i},y_{i})-z\left(x_{i+v},y_{j+p}\right)\right]}^{2}\right\}}^{q}\;$$

Then, the generalised variogram sill was calculated and compared for metastatic/non-metastatic samples. The small order moments, 0<q<2, are responsible for the core of the probability density function (PDF), whereas higher moments contribute to the tails of the PDF. For q=1, the generalised variogram is the empirical variogram. Comparing generalised and simple variogram sills of metastatic/non-metastatic samples led to clear differentiation. Theta statistics [46], rescaled range, surface, and phase and monofractal analysis are presented in Supplementary Materials and Methods S1–S5.

## 3. Results

### 3.1. Optical and AFM Microscopy of CRC Histological Sections

**Figure 1:**
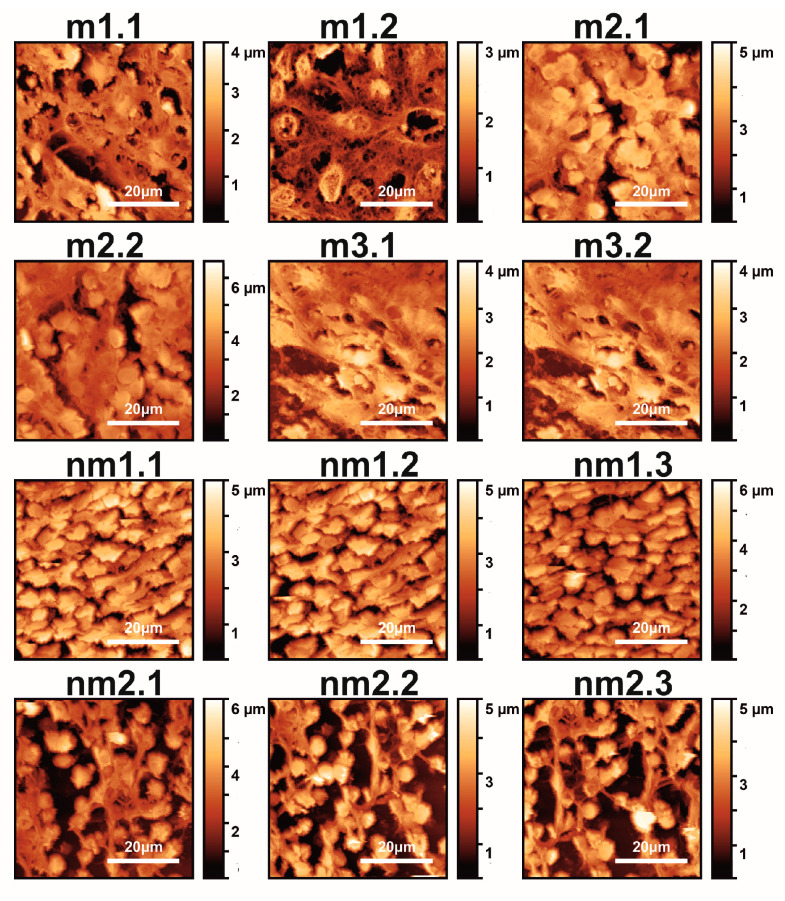
AFM images (12 images shown out of 18 total) of metastatic (m1.1–m3.2) and non-metastatic (nm1.1–nm2.3) human CRC histological sections. The first and second numbers refer to the patient and sample, respectively (m and nm belong to five patients). The colour bar in the vertical *y*-axis represents the *z*-heights of the image area.

Typical AFM CRC metastatic and non-metastatic histological tissue images extracted during 2021 from five patients are shown in Figure 1.

**Figure 2:**
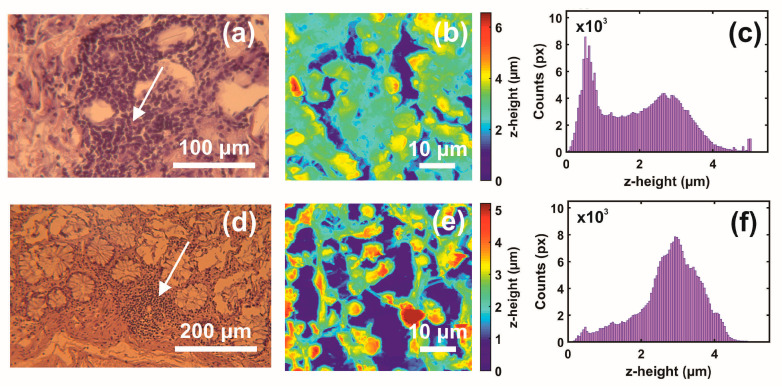
Optical and AFM images and *z*-height distribution of human CRC histological metastatic/non-metastatic sections. (**a**) Optical image (40×) of a metastatic section (0.17 mm × 1.73 mm). (**b**) AFM image of metastatic tissue at the specified point of the image in (**a**) (arrow). (**c**) The *z*-height distribution from the AFM image shown in (**b**). (**d**) Optical image (20×) of non-metastatic histological section. (**e**) AFM image of non-metastatic tissue at the specified point of the image in (**d**) (arrow). (**f**) The *z*-height distribution from the AFM image shown in (**e**).

The first and second indexing numbers are associated with the patient and sample parts. The visual differentiation between the metastatic and non-metastatic tissues in AFM images is unclear. On the contrary, optical images (4×, 20×, 40×) of hematoxylin/eosin-stained CRC histological sections unveil metastatic/non-metastatic differentiation, as shown in Figure 2. The cells of metastatic tissues, as shown in Figure 2a–b, were closely spaced compared to the non-metastatic ones, shown in Figure 2d–e. Nevertheless, optical microscope differentiation between metastatic and non-metastatic cells might be subjective, as can be also seen, in the similarity of the corresponding histograms Figure 2c,f, and, in some cases, dependent on the operator.

### 3.2. Variograms of Gaussian Filtering Residuals 

**Figure 3:**
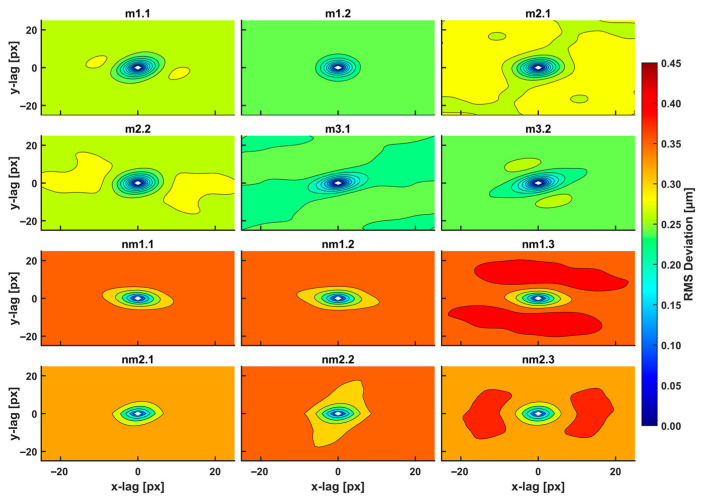
2D RMS deviation spectra of metastatic/non-metastatic CRC histological sections of the AFM images (12 images shown out of 18 total), Figure 2. The spectra were taken with a 3D Gaussian high-pass filter. The RMS deviation images represent a statistical measure of the deviation of heights within an area at a particular scale. The plot shows ellipse-like contours for small scales (100 nm–1 μm, 1–10.0 px) of equal-value RMS deviations for a given colour. Different RMS deviations from the colour index are noticeable for metastatic/non-metastatic sections. Non-metastatic CRC sections are characterized by higher values of RMS deviation within the closed and open areas compared to non-metastatic ones.

The two-dimensional (2D) variograms of the residuals of the Gaussian-filtered AFM images in Figure 1 of metastatic and non-metastatic histological tissues, along with all directions and sustained closed elliptic and open contours, are shown in Figure 3.

**Figure 4:**
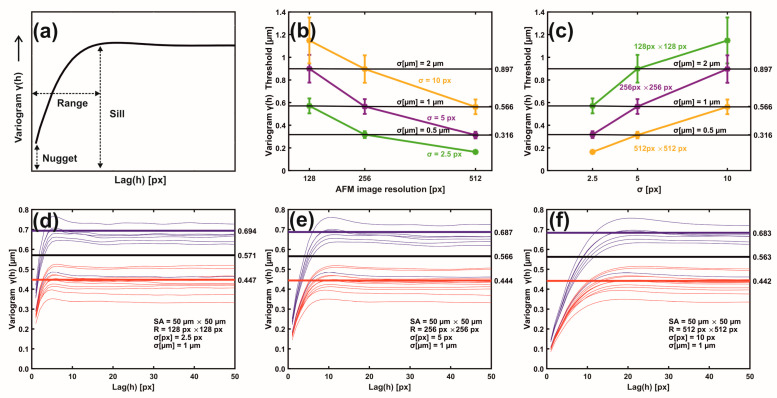
Metastatic/non-metastatic 1D variograms of residual Gaussian filtering. (**a**) A variogram’s typical parameters. (**b**) Metastatic threshold vs. AFM image resolution for different *σ* (px, μm) values. The metastatic threshold stays invariant for identical σ (μm) values. (**c**) Metastatic threshold vs. *σ* (px) at different AFM image resolutions. The metastatic threshold is invariant for identical σ (μm) values. (**d**) Variogram lines of metastatic (red) and non-metastatic (blue) groups. AFM image size 50 μm × 50 μm, resolution 128 px × 128 px, standard deviation σ (μm) = 1 μm, *σ* (px) = 2.5 px. The black sill line is the metastatic threshold defined as the median value of mean sill values of metastatic and non-metastatic tissues. Above the threshold line, tissues are non-metastatic, and they are metastatic below the line. Red and blue lines represent the mean metastatic/non metastatic sill values. (**e**) The same as (**d**) with image resolution 256 px × 256 px and *σ* (px) = 5.0 px. (**f**) The same as (**e**) with image resolution 512 px × 512 px, and *σ* (px) = 10.0 px.

The closed areas of the same colour characterise equal RMS deviations of small-size spatial scale differences (small lag vectors). Spatial correlations with dimensions below 0.5 μm originate from small biological and structural tissue topologies. For a Gaussian filter applied with standard deviation *σ* (px), the kernel box size along each axis is (6*σ* + 1) (px), and the lag vectors’ zero limits (nugget) is 1 px, Figure 4a. For the standard deviation *σ* of values between 2.5, 5.0, and 10.0 px, the magnitude of RMS deviation of closed contour areas diverges for metastatic and non-metastatic phases, as shown in Figure 3 and Supplementary Figure S1. Close to the centre of the 2D variograms, a relatively large RMS deviation is the typical signature of non-metastatic tissues. Colour indexing reveals that the mean RMS deviations of the metastatic and non-metastatic tissues are ~0.17 and ~0.27 μm, respectively (arctic blue and lemon yellow, respectively, in Figure 3). Therefore, the RMS deviation of the non-metastatic phase is noticeably more prominent than the metastatic one. The differentiation between metastatic and non-metastatic tissues was also retained for lower resolutions images of equal size (50 μm × 50 μm), e.g., for 256 px × 256 px and 128 px × 128 px image sizes and for values of *σ* between 2.5 and 10.0 px, as detailed in Supplementary Figure S1.

The 2D metastatic and non-metastatic variograms were comprehensively interpreted and quantified with 1D variograms, as seen in Figure 4a–f. The amplitude of lag vectors for all directions is along the *x*-axis. Along the *y*-axis, the non-overlapping sill values γ(h) of metastatic (red curves) and non-metastatic (blue curves) histological tissues represent lag vectors of zero correlation, with a relatively wide gap between the sill values of the two histological groups. 

In addition, the sill values of non-metastatic tissues are constantly placed above the sill values of metastatic ones, as seen in Figure 4d–f. Most importantly, for different image resolutions (pixels per line) and the same σ (μm), the sill indexes were invariant for the metastatic and non-metastatic groups, as seen in Figure 4d–f and Supplementary Figure S2. The mean sill value of each metastatic and non-metastatic group (red and blue lines parallel to the *y*-axis) was extracted from the average sill values of the associated histological tissues, as shown in Figure 4d–f. The median value of the mean sill values of metastatic and non-metastatic tissues defines the threshold lines (black line), above which tissues are non-metastatic, and they are metastatic below the line. For different image resolutions and identical σ values equal to 1 μm, the set of three different pixels and *σ* pairs, (128 px × 128 px, 2.5 px (1 μm)), (256 px × 256 px, 5.0 px (1 μm)), and (512 px × 512 px, 10.0 px (1 μm)), retained almost constant threshold sill values equal to 0.571, 0.566 and 0.563 μm, respectively, as shown in Figure 4d–f.

Similarly, two sets of pixels and identical σ (μm) values, ((128 px × 128 px, 5 px (2 μm) and (256 px × 256 px, 10.0 px (2 μm)) as well as ((256 px × 256 px, 2.5 px (0.5 μm) and (512 px × 512 px, 5.0 px (0.5 μm)), retained almost the same threshold sill values equal to 0.899 and 0.896 as well as 0.318 and 0.314 μm, respectively; this is also shown in Supplementary Figure S2. Variograms of low-resolution images and large *σ* values bore wider gaps and high uncertainty between the mean sill values of metastatic and non-metastatic variogram groups (bands), as shown in Supplementary Figure S2. Relatively large *σ* values amplified the uncertainty of information. The optimum metastatic differentiation for the current experimental configuration was obtained at a resolution of 512 px × 512 px and *σ* = 5.0 px. The threshold criteria for differentiating metastatic and non-metastatic tissues were successful in 17 out of 18 samples, the exception being sample nm2.4, which was non-metastatic but appeared to have metastatic behaviour. However, by applying higher moments than two (vide infra), the nm2.4 sample showed the correct non-metastatic behaviour.

### 3.3. Moments of Gaussian Filtering Residual Variograms 

**Figure 5:**
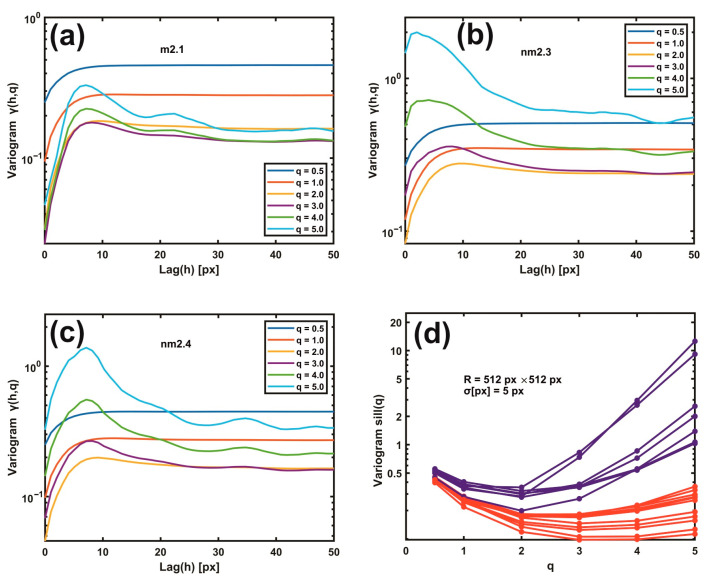
Gaussian filtering residual variograms of different moments q. (**a**) Gaussian filtering residual variograms of q moments from 0.5 to 5.0 for the metastatic tissue m2.1. (**b**) The same as (**a**) for the non-metastatic tissue nm2.3. (**c**) The same as (**b**) for the non-metastatic tissue nm2.4. Threshold criteria for differentiating metastatic and non-metastatic tissues, Figure 4, do not function for the sample nm2.4. (**d**) Gaussian filtering residual variograms of higher moments upsurge the differentiation between metastatic (red) and non-metastatic (blue) groups of lines. For higher moments than two, the nm2.4 tissue sample shows the correct non-metastatic state.

Gaussian filtering residual variograms of higher moments upsurge the differentiation between metastatic and non-metastatic AFM images. For large scaling exponents q, the difference between metastatic and non-metastatic tissues widens further than the lower q values, as seen in Figure 5a–d. For example, the variogram sill value (512 px × 512 px, *σ* = 5.0 px) for q > 3 is always higher than q < 3 in all non-metastatic samples compared to the metastatic ones, as seen in Figure 5a–d and Supplementary Figure S3. Furthermore, the nm2.4 tissue sample, the unsuccessful exception in the 1D variograms’ threshold criterion that behaves as a metastatic one, now adopts the correct non-metastatic behaviour for higher moments (q > 2), agreeing with the pathologist’s examination. However, for different image resolutions and Gaussian filtering *σ* values, the moments that give the corrected result for the nm2.4 tissue deviate, as shown in Supplementary Figure S4. Therefore, the threshold criterion of metastasis varies between different moments. Consequently, metastatic differentiation improved at higher moments.

### 3.4. Theta Statistics

**Figure 6:**
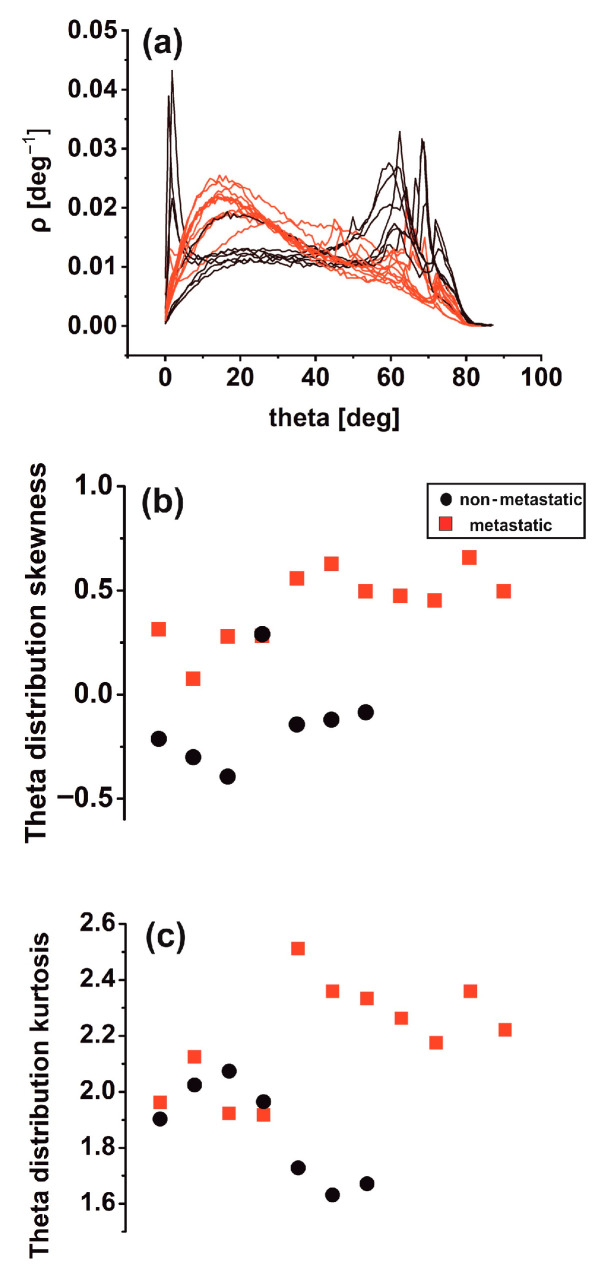
Theta statistics of eighteen metastatic/non-metastatic CRC histological sections. (**a**) Theta spectra of metastatic (red)/non-metastatic (black) sections. The metastatic sections have one maximum value at 15°. The non-metastatic sections have two maxima, the first at small angles (2°) and the second at high angles (>60°). (**b**) Theta distribution skewness of metastatic/non-metastatic CRC histological sections with negative and positive skewness values. One metastatic and non-metastatic point almost coincide. (**c**) Theta distribution kurtosis of metastatic/non-metastatic CRC histological sections.

Differences in theta distribution profiling [46] may be critically associated with biological interactions between metastatic tumour cells and the ECM, leading to tissue differentiation. Other surface roughness characteristics in metastatic tissues (11 tissue samples) led to notably broader inclination angle distributions than the non-metastatic ones (seven tissue samples). Sharp peaks in the theta distribution diagram characterise the last. It appears that non-metastatic tissues are typified by structural surface regularity, which is highlighted by the sharp peaks at higher theta values in Figure 6a. In contrast, random patterns and de-oriented structures define the metastatic phase. Skewness and kurtosis are differentiating measures in theta distribution. The skewness of theta distribution of all metastatic sample AFM images, seen in Figure 6b, was positive, thereby agreeing with Figure 6a.

In contrast, the skewness of non-metastatic tissues was negative (except for one sample), owing to the sharp peaks on the right side of the graph. In addition, the kurtosis of theta distribution deviated from zero in all AFM images for metastatic and non-metastatic samples, leading to non-normal distributions as expected, as shown in Figure 6c. Although not in all cases, the skewness and kurtosis of metastatic tissues tended to have relatively large values.

### 3.5. Surface Analysis

The standard statistical parameters of stained CRC histological sections of AFM images were calculated; the details are shown in Supplementary Figure S5a–b. The *z*-height distribution values of the AFM images of metastatic CRC histological tissues appear to have a wider dispersion around a mean value and obtain far more extreme values than the non-metastatic ones; the details are shown in Supplementary Figure S5a. In addition, the RMS roughness values of metastatic tissues, represented by red squares, are smaller than the circular black values for non-metastatic ones; the details are shown in Supplementary Figure S5b. Contrary to 1D and 2D variograms and theta distribution, surface analysis did not clearly distinguish between metastatic and non-metastatic phases. However, there was an underlying tendency towards lower roughness values for metastatic tissues compared to non-metastatic ones, which is in agreement with the results from variograms and theta analysis shown in Figure 3 and Figure 4.

### 3.6. Rescaled Range Analysis (Hurst Exponent)

Rescaled range analysis/surface statistics were also applied along the same direction for 512 lines of each tissue image; the details are shown in Supplementary Figure S5c–e. First, each of the 2D AFM images was transformed into a 1D array by putting every line of 512 px one after another, and the Hurst exponent of each 512 px × 512 px array string was calculated, as shown in Supplementary Figure S5c. The same analysis was also performed for every line of each AFM image. Then, the mean value of the Hurst exponent of each AFM image was calculated for all lines, and the histogram was plotted; this is shown in Supplementary Figure S5d.

The Hurst exponents and their trends as extracted from the two algorithms were dissimilar; this is shown in Supplementary Figure S5c–d. The differentiation is expected because the two methods bear different correlations and connectivity between lines. The distribution histogram of the Hurst exponent distributed between the 512 lines is shown in Supplementary Figure S5e. There was considerable variation of the Hurst exponent when changing the number of lines. The differentiation between metastatic and non-metastatic tissues is unclear, despite shifting the distribution function to the right relative to the maximum mean value for the metastatic tissues. The Hurst exponent does not differentiate between metastatic and non-metastatic tissues. Rescaled range analysis as second-order statistics usually provides insights for monofractal systems. However, metastasis is a dynamic process that drives cancer to higher non-reversible hierarchical levels.

### 3.7. Phase Analysis

Because the phase images correlate with the topographical ones, the signal’s driving frequency is associated with a phase shift owing to adhesion, stiffness, or friction. Therefore, the standard statistical phase parameters of the stained CRC histological sections’ AFM images were calculated. As for the AFM amplitude imaging, the *z*-height distribution of non-metastatic tissues had a broader dispersion around the mean value; this is shown in Supplementary Figure S6a. In addition, the RMS roughness values of metastatic tissues were lesser than for non-metastatic ones; this is shown in Supplementary Figure S6b. Again, there is no clear distinction between metastatic and non-metastatic tissues. Rescaled range analysis was also applied for phase images; the Hurst exponent, shown in Supplementary Figure S6c–d, and the Hurst exponent distribution were extracted between the phase lines, which are shown in Supplementary Figure S6e.

As for the amplitude images of the Hurst exponent, the differentiation between metastatic and non-metastatic tissues is unclear. However, surface and rescaled analysis bear noticeable similarities despite limiting metastatic information.

### 3.8. Monofractal Image Analysis 

Monofractal dimensionality (D_f_*)* of metastatic and non-metastatic tissues [28] was calculated by using cube counting, triangulation, power spectrum, and partition algorithms, as shown in Supplementary Figure S7. The cube counting and the triangulation methods, which are detailed in Supplementary Figure S7a,d, provided lower D_f_ numbers for metastatic tissues than the other two methods, which are shown in Supplementary Figure S7b–c, where the fractal dimensionality of non-metastatic tissues is relatively more minor than that of metastatic ones. Overall, the four algorithms have no clear differentiation between metastatic and non-metastatic tissues. 

## 4. Discussion

### 4.1. The Intricacy of the Cancer Problem

Cancer is a multivariate and complex disease, and despite intense research starting as early as the last century, it still represents a challenging issue. There are many reasons for this. Over the years, clinical methods applied favourable average practices. Nevertheless, cancer is highly heterogeneous even within the same cell and in similar classes. Consequently, an overall positive average outcome does not translate to individual positive results.

Moreover, despite the significant effort and the enormous resources devoted to cancer research, it is still unknown why some drugs are more effective for some individuals than others. Besides other critical issues, deciphering cancer growth, metastatic progression, and migration at the nanoscale is vital for survival [47]. Likewise, metastasis shapes one of the six crucial hallmarks of cancer [2]; the others include sustaining proliferating signalling, evading growth, suppression, and activating invasion. The intricacy is further increasing because almost 12 years after the seminal paper of Hanahan and Weinberg [2], four additional cancer hallmarks highlight the disease’s complexity, signalling that traditional approaches need new strings in their bows [48]. First, it is now well understood that a novel interdisciplinary approach to the cancer menace is required, one where biology, physics, and mathematics, in an integrated step, could illuminate the dark pathways of cancer progression or even discover hidden physical laws of the phase transition between healthy and carcinogenic cells. Second, even if critical sporadic and uncorrelated contributions to cancer research were made from different physics and cell biophysics fields, their integration is still intermittent in cancer research. Third, the metastatic phase is usually clinically validated with biomarkers. So thought, even when the diagnosed metastatic phase is discovered with an optical histological examination of spatial resolution less than 500 μm, it represents a late stage. Fourth, the multistep process of invasion and metastasis mimics, under certain circumstances, a developmental process referred to as the Epithelial–Mesenchymal Transition (EMT) [49,50]. EMT is a functional process that allows a polarised epithelial cell, which normally interacts with basement membrane and other epithelial cells, to undergo multiple biochemical and genetic changes that enable it to assume a mesenchymal cell phenotype, which includes a thin and elongated shape, enhanced migratory capacity, invasiveness, elevated resistance to apoptosis, and significantly increased production of ECM components [49]. The occurrence of a regulated reverse process, the Mesenchymal–Epithelial Transition (MET) [49], which involves the conversion of mesenchymal cells to epithelial derivatives, indicates a significant difference with the phenomenon presented by carcinoma cells during invasion and metastasis. Indeed, upon genetic deregulation of the structural and regulatory factors linked to the epithelial phenotype during pathological EMT, carcinoma cells can concomitantly acquire multiple attributes that enable invasion and metastasis because MET rarely happens in a controlled way in the primary tumour tissue. Nevertheless, once seeded in distant tissues, metastatic cells may partially or entirely activate MET pathways, enabling a few or even a single metastatic cell to grow into a metastatic mass [51].

Therefore, the analysis of cell and tissue architectures may represent a method for assessing the metastatic potential of cancer cells. Furthermore, the detection of EMT or mesenchymal cells in primary CRC tissues, which may improve their metastatic prognostic value, is possible because several marks associated with both mesenchymal and epithelial phenotypes are known [52]. However, these analyses involve complex molecular biology exams, such as tissue microarrays, RNA and protein expression, immunohistochemistry, and others, targeting multiple factors. Therefore, these exams’ complexity and high cost impair their evaluation in standard clinical laboratories. Contrary to optical imaging of colorectal cancer tissues with an image resolution of ~1.4 μm, as detailed in Supplementary Figure S8, the basic idea of the work is to identify carcinoma cells in the primary tumour sites with early morphological changes, which can indicate the activation of the EMT process. Indeed, during EMT, the carcinoma cells lose their cell–cell junctions and move apart, generating tiny but significant histological and cytological changes detected only at the nanoscale level with AFM with 97.7 nm image resolution, as shown in Supplementary Figure S9.

### 4.2. Variograms and Theta Statistics: Diagnostic Tools for Early Cancer Metastasis

The sill variogram values of metastatic CRC histological tissues from AFM image processing for the three patients in this study were below 0.566, the threshold line for image resolutions 512 px × 512 px and *σ* = 10.0 px (1 μm). For different configurations of image resolutions and σ values, the metastatic threshold line could be adjusted accordingly. The metastatic threshold line from variograms between the metastatic and non-metastatic phases defines the borderline between patients’ death and extended survival. Importantly, in the case of ambiguity, regarding the nm2.4 tissue, higher moments than 2nd-order variograms remove any possible mixing between metastatic and non-metastatic tissues, as shown in Figure 5 and Supplementary Figures S3 and S4. Contrary to the variograms and theta-statistics, the *p*-value statistics verify that the rescaled range, surface, phase, and monofractal analysis do not distinguish between metastatic and non-metastatic tissues, and the correlation between metastasis and tissue mono-fractality is vague, as shown in Supplementary Analysis S6’s two-sample t-test analysis and in Supplementary Tables S1–S3. Indeed, during the transformation of single premalignant cells into cancerous ones [53], the fractal dimensionalities do not necessarily imply the existence of fractal geometrical features. However, the rational interpretation of variograms, theta statistics, and multifractal analysis [54] revealed unforeseen but significant outcomes.

The sill values of primary tumour tissues that did not result in metastasis are higher than those of cells triggering metastases, indicating a high dispersion of residuals within the Gaussian filtering of *z*-heights along the *x*–*y* cell surface that is the signature of a complex biological structure. It is expected that tissues with metastatic cells should bear lower sill values due to high cell mobility and plasticity, ensuing small cell adhesion values. Indeed, based on the EMT model, in primary tumour tissue, carcinoma non-metastatic cells still present a degree of interactions through desmosomes, hemidesmosomes, and tight and adherent junctions (epithelial phenotype) that make cell and, consequently, tissue structure complex, as both have large sill values. On the contrary, metastatic cells, having lost the majority, or even all, connections with other cells and the ECM, have comparably lower sill values. Translating these observations into biological terms, we can affirm that in the primary tumour tissue, non-metastatic cells still present an epithelial phenotype along with an array of membrane proteins such as cadherins and integrins specialised in establishing connections with the ECM; their sill values are higher.

Conversely, based on the EMT model, the primary tumour tissue’s metastatic cells lost almost all ECM connections along with the corresponding membrane proteins (e-cadherin, integrins, etc.). As a result, they shifted their phenotype towards mesenchymal cells, presenting a smoother surface that lowered their sill score. Theta statistics also confirm this conclusion. Interpreting these results into biological terms, we can assume that the presence of epithelial cells, their adhesion receptors, and cell-cell and cell-ECM interactions increase the theta distribution’s skewness. On the contrary, mesenchymal cells deprived of several membrane proteins, relatively free to move and able to digest the ECM, present surfaces with reduced amounts of roughness, which translates into a lower theta distribution skewness.

By applying *p*-value statistics in second-moment variograms and the null hypothesis that the mean sill values of metastatic and non-metastatic tissues are the same, the differentiation between metastatic and non-metastatic tissues (*p*-value) is statistically significant with a probability of 99.99999%. The differentiating confidence for higher-than-2nd-order variogram moments for metastatic and non-metastatic tissues is further improved. High-order variograms of Gaussian residual filtering distinguish metastatic and non-metastatic tissues by categorising a well-defined threshold. The reason is that Gaussian filtering differentiates *z*-height features with sizes less than 97.5, 194.0, and 388.0 nm (for image resolutions 512 px × 512 px, 256 px × 256 px, and 128 px × 128 px, respectively). This result agrees with previous work [53], where microvilli, microridges, and the glycocalyx are responsible for the pericellular brush surface geometry structure. AFM imaging includes information from the cell’s surface, random cell volume cross-sections, CRC histological tissue encloses and ECM. Therefore, Gaussian filtering differentiates small biological features between metastatic and non-metastatic phases in the CRC–ECM system.

### 4.3. Cancer as a Dynamical Hierarchical Issue and Problem

In addition to the practical utility of variograms in cancer prognosis, grouping the well-defined threshold sill lines for metastatic and non-metastatic CRC tissues has broader implications in cancer research. Undeniably, the differentiation between the metastatic and non-metastatic phases defines two hierarchies in the CRC cell–ECM system. Generally, dynamical systems, such as cancerous ones, have structural (hardware) and functional (software) connotations that form ensembles of successfully interacting nested sets and subunits of variables and parameters. In addition, as the complexity of structural and functional systems depends on the number of their components and interconnections, complexity is inversely proportional to stability and degrees of freedom. Thus, it defines a particular hierarchical state (level). Furthermore, the systems afford a specific state–space–time description with certain collective properties (e.g., statistical moments, convolutions, distribution functions, and memory). From that state, during the evolution process across the dynamical paths, the systems within “limited-time series” are commonly driven to lower complexities with fewer degrees of freedom and, thus, to more stable states (high viability).

The dynamical systems evolve from lower hierarchical levels with many degrees of freedom and high complexities to higher hierarchical levels with fewer degrees of freedom and lower complexities. Besides structural hierarchies, the systems are characterised by the formation dynamic. The higher levels receive selective information from the lower levels through the cognition (memory) [55] of collective properties. In turn, they exercise negative feedback control commands on the dynamics of the lower levels in their effort to occupy successfully higher hierarchical levels. Therefore, interactive systems are characterised by mutual “simulation”. One dynamic system, say, a non-metastatic one, tries to simulate another with fewer degrees of freedom and higher stability (metastatic system). Thus, a non-metastatic system will eventually occupy higher hierarchical levels of lower complexity with higher stability. The opposite route, the evolution from higher hierarchical levels to lower ones, requires the expenditure of additional information energy (entropy). Therefore, in most cases, the reverse process is energetically unfavoured. Along these lines, the selective differentiation between metastatic and non-metastatic groups evinces the dynamic evolution of hierarchical carcinogenic states during disease progression [56]. This advancement ranges from lower carcinogenic hierarchical levels of higher complexity and low stability (premalignant conditions) to higher ones that are less complex and stable (metastatic forms). The heterogeneous chemotherapy results might explain the one-way evolution dynamic and the non-reversibility and interchangeability of hierarchies. If the hierarchical dynamic is deciphered, then cancer’s therapeutic protocols and their road maps might change, as the two hierarchies should require different therapeutic protocols. Within the above framework, perhaps, the unknown efficacy of next-generation non-polar magnetic nanoparticles [57], functionalised with biodegradable and biocompatible polymers [58], might have a positive effect on external cellular membrane functionality of human CRC metastatic cells and histological sections [59]. 

## 5. Conclusions

The work presents a novel methodology for early nanosized (97.7 nm) identification of metastasis from primary colorectal cancer histological tissues by processing AFM cancer tissue images using second-order or higher variograms and theta statistics. Moreover, the image processing algorithm includes rescaled range analysis, average *z*-height, RMS roughness, phase spectra, and monofractal image analysis. Five patients, three metastatic and two non-metastatic, and eighteen AFM images of histological cancer samples, eleven metastatic and seven non-metastatic, were used in the study. The sill values of variograms and theta distribution skewness identify metastasis with 99.99999% and 99.99% confidence (*p*-value), respectively. Variogram and theta statistics as well as metastatic differentiation, set different irreversible hierarchical and complexity levels for the metastatic progression dynamic.

## Data Availability

All data and software codes of this study are available from the corresponding author upon request.

## Supplementary Materials

The following supporting information can be downloaded at: https://www.mdpi.com/article/10.3390/cancers15041220/s1, Analysis S1: Theta Statistics [46,60]; Analysis S2: Rescaled Range Analysis [61,62]; Analysis S3: Surface Statistics; Analysis S4: Phase Spectra; Analysis S5: Monofractal Image Analysis; Analysis S6: Two-Sample t-test Analysis; Figure S1: 2D variograms of the residuals of the Gaussian filtered AFM images of 16 out of 18 metastatic and non-metastatic histological tissues; Figure S2: Non-metastatic (blue lines) and metastatic (red lines) 1D variograms Variograms of low-resolution images and large σ’s bear wider gaps and high uncertainty between the mean sill values of metastatic and non-metastatic variogram bands; Figure S3: Gaussian filtering residuals variograms of different moments (q); Figure S4: Variogram sill values for different image resolutions and Gaussian filtering σ vs. scaling exponents q for metastatic (red) and non-metastatic (blue) tissues; Figure S5: Standard surface statistical parameters and rescale range analysis/surface statistics of AFM images of CRC histological sections; Figure S6: Surface statistical phase spectra of CRC metastatic (red squares) and non-metastatic (black circles) tissue AFM images; Figure S7: Fractal dimension D_f_ of images of CRC metastatic (red squares) and non-metastatic (black circles) tissue AFM images were calculated with four different methods; Figure S8: Optical images of metastatic and non-metastatic tissue sections captured with 4× (d, h, j, k, l, m, and o), 10× (a and e), and 40× (b, c, f, g, i, and n) magnification; Figure S9: Typical zoomed scanned areas 10 µm x 10 µm of AFM images of Fig.1 of metastatic (m1.2&m2.1) and non-metastatic (nm2.2&nm2.3) human CRC histological sections; Table S1: Variogram parameters (sill and range), mean value and threshold, the arithmetic means of sill values of metastatic (M) and non-metastatic (NM) tissues, with and without the nm2.4 sample (red colour), for different AFM image resolutions and standard deviation σ values in px and μm, respectively; Table S2: Statistical parameters of sill values of non-metastatic (NM) and metastatic (M) tissues for different Gaussian filter sigma *σ* values (px). Μean sill values, standard deviation (StDev), standard error of mean (SE mean), the difference of M and NM mean values (D), standard deviation of difference (StDev of D), 95% confidence interval for the difference (95% CI for D), threshold ± 95% CI for D, p-value statistic parameter for the hypothesis NM. ≠ M; Table S3: Statistical parameters of theta distribution skewness (Figure 6b) and kurtosis (Figure 6c), average *z*-height (nm) (Figure S5a), RMS roughness (nm) (Figure S5b), mean Hurst exponent of 512 lines (topography) (Figure S5c), Hurst exponent of 512 lines as one line (topography) (Figure S5d), phase average value (V) (Figure S6a), phase “RMS roughness” (V) (Figure S6b), mean Hurst exponent of 512 lines (phase) (Figure S6c), Hurst exponent of 512 lines as one line (phase) (Figure S6d), fractal dimension box counting (Figure S7a), partitioning (Figure S7b), triangulation (Figure S7c) and power spectrum (Figure S7d) methods of non-metastatic (NM) and metastatic (M) tissues. Μean values, standard deviation (StDev), standard error of mean (SE Mean), the difference of M and NM mean values (D), standard deviation of difference (StDev of D), 95% confidence interval for the difference (95% CI for D), *p*-value statistic parameter for the hypothesis NM. ≠ M.
