# Supplementary material for: Nanoscale Prognosis of Colorectal Cancer Metastasis from AFM Image Processing of Histological Sections
Source: Cancers (Basel). 2023 Feb 14;15(4):1220. doi: 10.3390/cancers15041220 (PMC9953928; doi:10.3390/cancers15041220)
Supplement: Supplementary file 1 [file cancers-15-01220-s001.zip › cancers-2175546-supplementary.pdf]

Supplementary Material

Title: Nanoscale prognosis of colorectal cancer metastasis from AFM image processing of histological sections

Authors name:
Vassilios Gavriil[1], Angelo Ferraro[1], Alkiviadis-Constantinos Cefalas[1], Zoe Kollia[1], Francesco Pepe[2], Umberto Malapelle[2], Caterina De Luca[2], Giancarlo Troncone[2], and Evangelia Sarantopoulou[1*]

Affiliations:
1) National Hellenic Research Foundation, Theoretical and Physical Chemistry Institute, 48 Vassileos Constantinou Avenue, Athens, 11635, Greece
2) Dipartimento di Sanità Pubblica, Università degli studi di Napoli "Federico II", via Pansini 5, Napoli, 801301, Italy

Supplementary materials and methods

S1. Theta Statistics

Inclination-slope distributions were applied for metastatic and non-metastatic AFM images using the Gwyddion software [60]. The polar angle θ between the horizontal plane and the "central derivative plane" in every pixel is related to the surface-profile gradient $\vec{v} = \left(\frac{dz}{dx}, \frac{dz}{dy}\right)$ via the equation $\theta = tan^{-1}|\vec{v}|$. The polar angle θ is always positive and rises with a slope $\left|\frac{dz}{dx}, \frac{dz}{dy}\right|$ while the integral $\int \rho(\theta) d\theta, for\ \theta \in \left[0, \frac{\pi}{2}\right]$ is normalized to one and $\rho = \frac{Number\ of\ counts}{\int (Number\ of\ counts) d\theta}$. For quantified comparison between different slope distributions the skewness $Sk = \frac{1}{N\left(\sqrt{\frac{1}{N}\sum_{i=1}^{N}|X_i - \bar{X}|^2}\right)^3} \sum_{i=1}^{N}(X_i - \bar{X})^3$ and kyrtosis $Ku = \frac{1}{N\left(\sqrt{\frac{1}{N}\sum_{i=1}^{N}|X_i - \bar{X}|^2}\right)^4} \sum_{i=1}^{N}(X_i - \bar{X})^4$ of slope distribution is calculated [46].

S2. Rescaled Range Analysis

The Hurst exponent of every line of the AFM image was calculated using the rescaled range analysis of Hurst [61]. The algorithm for these calculations was designed and run in MATLAB. 9.4.0.813654 (R2018a), The MathWorks Inc.; Natick, MA, the USA based on Weron's algorithm [62]. Then, the mean value of the Hurst exponent of every AFM image was calculated, and the histogram was plotted. In addition, the 2D AFM image was transformed into a 1D array by putting every line after another. Finally, the Hurst exponent of every 1D AFM image was calculated with the same methodology. The same analysis was also performed on "phase" images.

S3. Surface Statistics

Several different parameters were used for the first qualitative evaluation of surface characteristics of metastatic and non-metastatic samples. First, was calculated the average z-height (nm) arithmetic mean defined as the sum of all height values divided by the number of data points $\bar{Z} = \frac{1}{N}\sum_{1}^{N} Z_i$. Next, the RMS roughness was calculated, which is the square root of the mean value of the squares of the distance of the points from the image mean value, $R_{rms} = \sqrt{\frac{1}{N}\sum_{1}^{N}(Z_i - \bar{Z})^2}$.

S4. Phase Spectra

The AFM tapping mode generates the phase images, and the signal's frequency (phase) is a function of the driving frequency adjusted to be at the actual probe resonance, as it is shifted due to the tip-sample forces. Tip height variation of phase images correlates with the topographical ones. The signal's driving frequency is associated with a phase shift owing to adhesion, stiffness, or friction, and the interaction between tip and surface will cause lag oscillation. The RMS phase roughness, mean phase, mean phase Hurst exponent of 512 lines, and Hurst exponent of 1D image vector was calculated for all phase AFM images.

Statistical analysis was applied to both images. The average phase shift *(V)* is an arithmetic mean defined as the sum of all height values divided by the number of data points $\bar{P} = \frac{1}{N}\sum_{1}^{N} P_i$. The RMS roughness is the square root of the mean value of the squares of the distance of the points from the image mean value, $R_{rms} = \sqrt{\frac{1}{N}\sum_{1}^{N}(P_i - \bar{P})^2}$.

S5. Monofractal Image Analysis

The self-affine properties in a specific range of scales were analyzed using monofractal analysis. Four methods calculated the fractal dimension D_f using "Gwyddion, SPM data visualization and analysis tool." The methods used to calculate D_f are cube counting, triangulation, and partition. Cube counting arises from the definition of box-counting fractal dimension where an *l* cubic lattice constant superimposes on the *z*-expanded surface. First, *l* is set at *X/2* (*X* is the surface's edge length), providing a lattice of 2^3 cubes *N(l)*, containing at least one pixel. The lattice constant *l* is reduced stepwise by a factor of two, and the process repeats until *l* equals the distance between two adjacent pixels. The slope of a plot of *log(N(l))* versus *log(1/l)* gives D_f. Triangulation is similar to the previous method. A grid of unit dimension *l* is placed on the surface, defining the location of several triangle vertices. For *l* = *X/4*, 32 triangles of different areas are inclined at different angles, with the *xy* plane covering the surface. The areas of all triangles are calculated and summed to approximate the surface area *S(l)* for a given *l*. Next, the grid size decreases by a successive factor of 2, and the process continues until *l* equals the distance between two adjacent pixel points. The slope of a plot of *log(S(l))* versus *log(1/l)* is the number $D_f - 2$. The partitioning algorithm is based on the scale dependence of the variance of fractional Brownian motion. One divides the entire surface into equal-sized squared boxes, and the variance (power of RMS heights) was calculated for the particular box size. The slope value β of a least-square regression line fits the data points in the log-log plot of variance extracts D_f's as $D_f = 3 - \beta/2$.

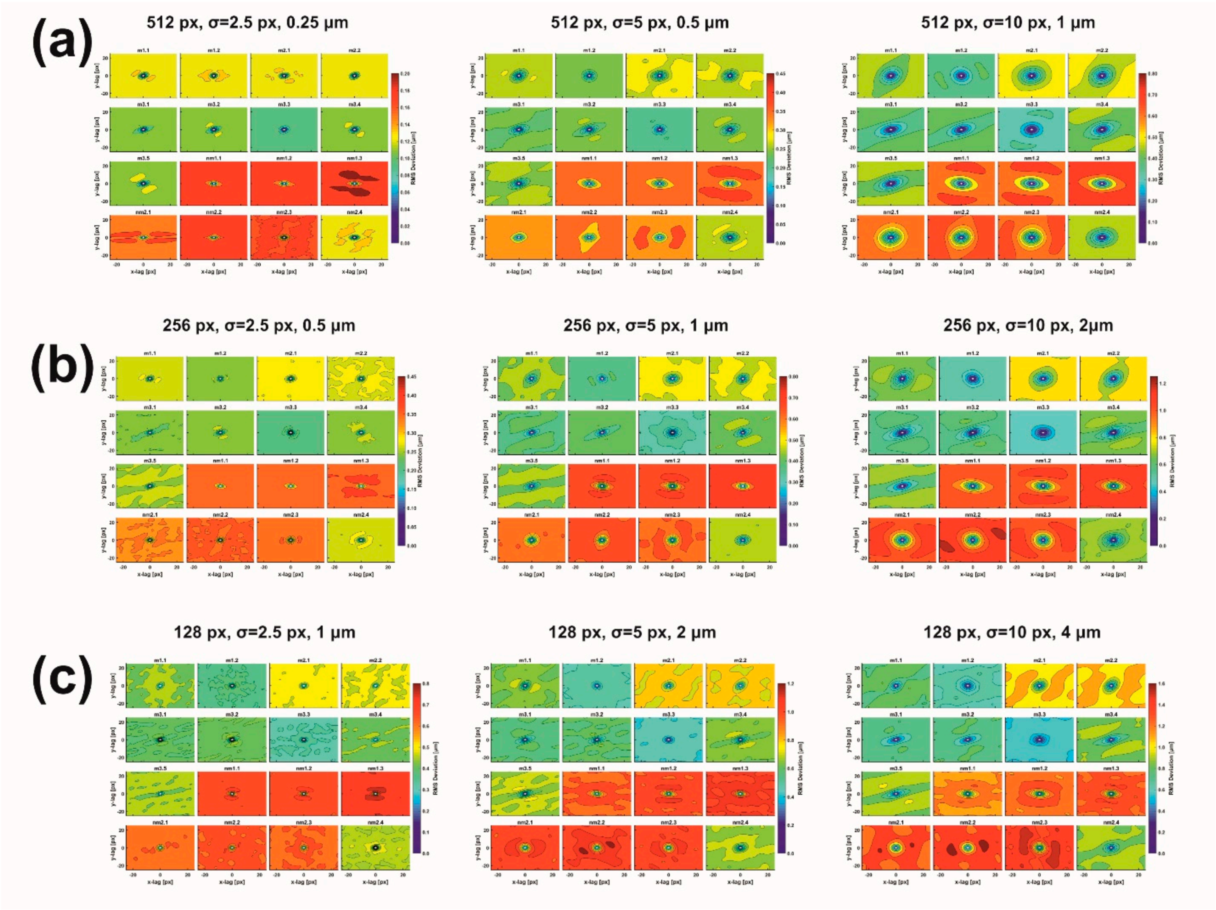

Figure S1. 2D variograms of the residuals of the Gaussian filtered AFM images of 16 out of 18 metastatic and non-metastatic histological tissues. **(a)** AFM image resolution 512 px x 512 px, and σ 2.5, 5.0 and 10.0 px. **(b)** AFM image resolution 256 px x 256 px, and σ 2.5, 5.0 and 10.0 px. **(c)** AFM image resolution 128 px x 128 px and σ 2.5, 5.0 and 10.0 px. The magnitude of RMS deviation of closed contour areas diverges for metastatic and non-metastatic phases except for sample nm2.4, which shows metastatic behaviour. The sample nm2.4 attains the correct metastatic state for higher moments (q>2).

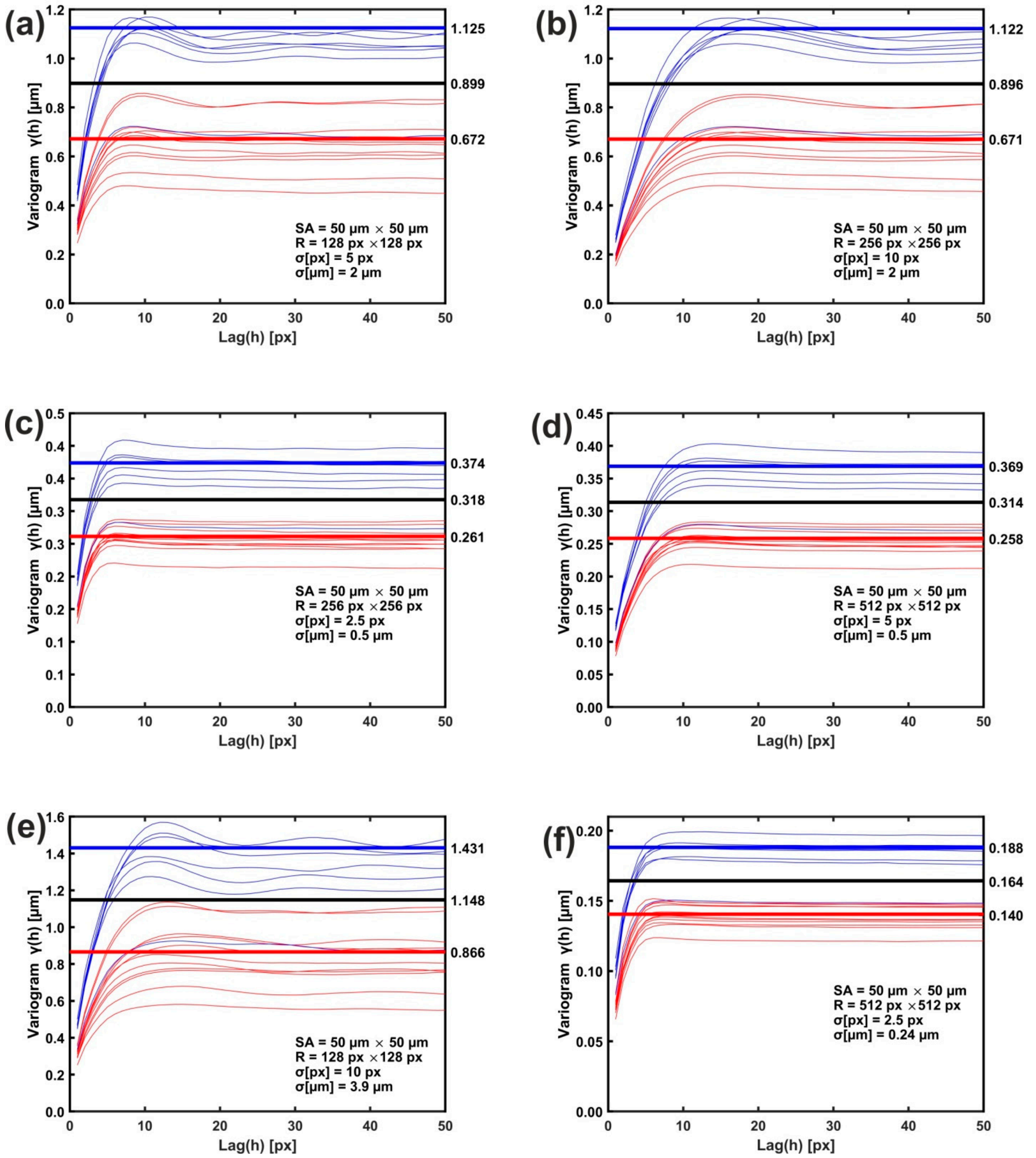

Figure S2. Non-metastatic (blue lines) and metastatic (red lines) 1D variograms. Variograms of low-resolution images and large *σ's* bear wider gaps and high uncertainty between the mean sill values of metastatic and non-metastatic variogram bands. **(a,b)** The pair 128 px x 128 px, *σ* 5.0 px (2 μm) and 256 px x 256 px, *σ* 10.0 px (2 μm) hold the same metastatic threshold values. **(c,d)** The pair 256 px x 256 px, *σ* 2.5 px (0.5 μm) and 512 px x 512 px, *σ* 5.0 px (0.5 μm), hold the same metastatic threshold values. **(e,f)** The pair 128 px x 128 px, *σ* 10.0 px (3.9 μm) and 512 px x 512 px, *σ* 2.5 px (0.24 μm) hold the same metastatic threshold values.

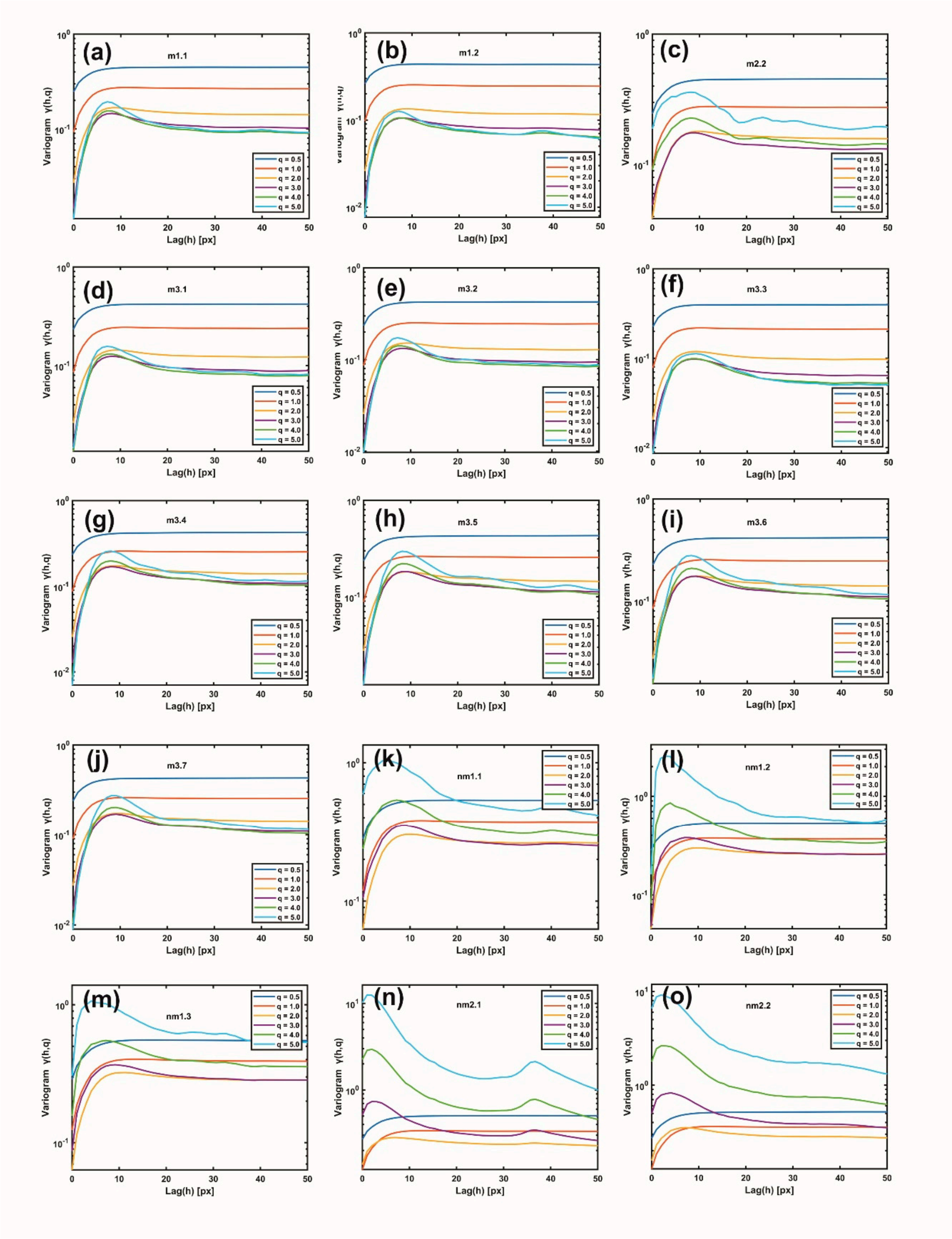

Figure S3. Gaussian filtering residuals variograms of different moments (q). **(a)-(j)** for metastatic tissues. **(k-o)** for non-metastatic tissues.

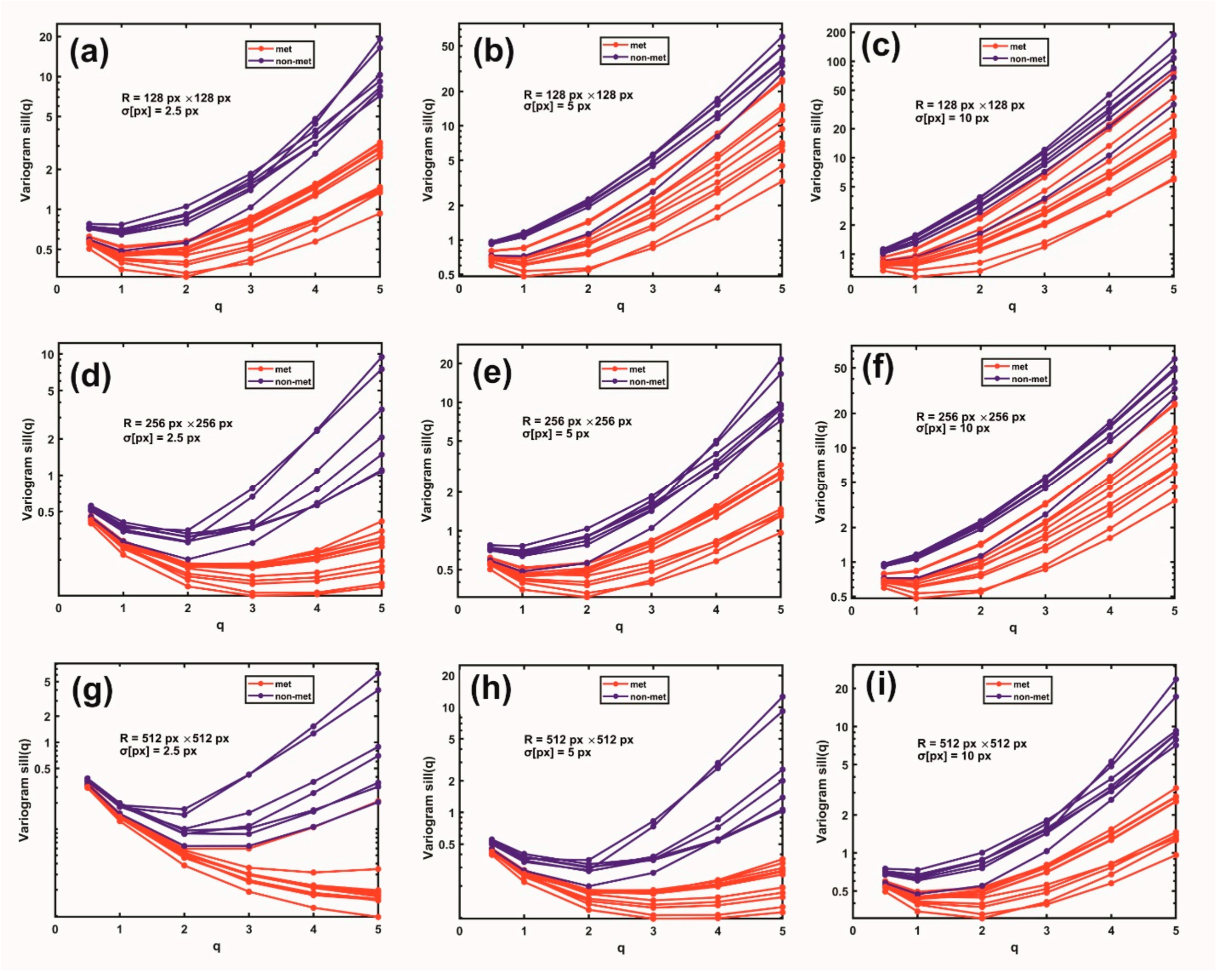

Figure S4. Variogram sill values for different image resolutions and Gaussian filtering σ *vs.* scaling exponents q for metastatic (red) and non-metastatic (blue) tissues. **(a-c)** 128 px x 128 px, σ 2.5, 5.0 px and 10.0 px. **(d-f)** 256 px x 256 px, σ 2.5, 5.0 and 10.0 px. **(g-i)** 512 px x 512 px, σ 2.5, 5.0 px and 10.0 px. The nm2.4 tissue, the non-successful sample, in the 1D variograms metastatic threshold criterion, performs as metastatic for higher moments (q>2), adopting the correct non-metastatic state in agreement with the subjective optical microscopic examination. The differentiation between metastatic and non-metastatic tissues is improved for high q values.

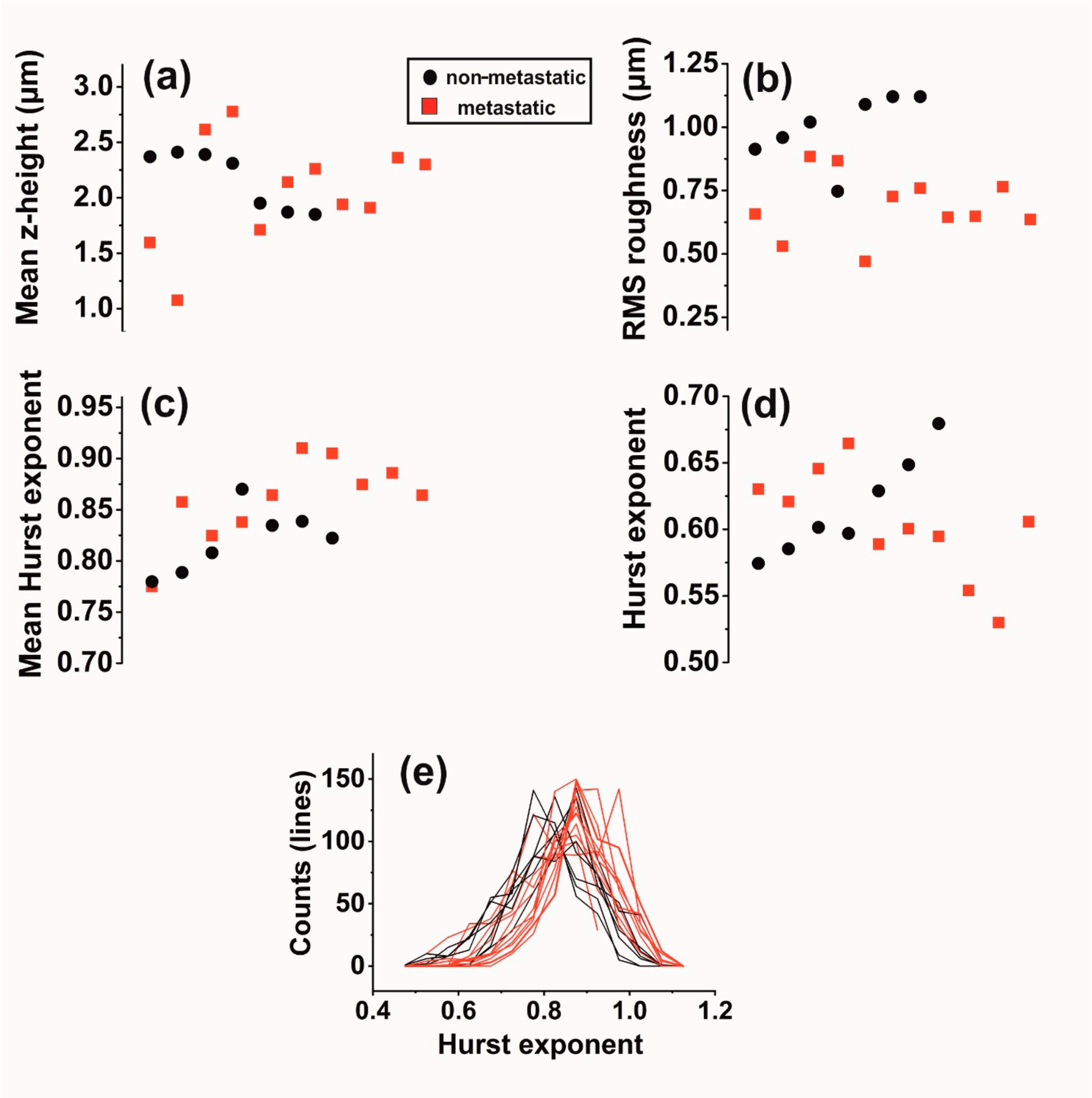

Figure S5. Standard surface statistical parameters and rescale range analysis/surface statistics of AFM images of CRC histological sections. **(a)** Mean *z*-height distribution. **(b)** RMS roughness. **(c)** Mean Hurst exponent. **(d)** Hurst exponent. **(e)** Hurst exponent distribution. The differentiation between metastatic and non-metastatic sections is unclear.

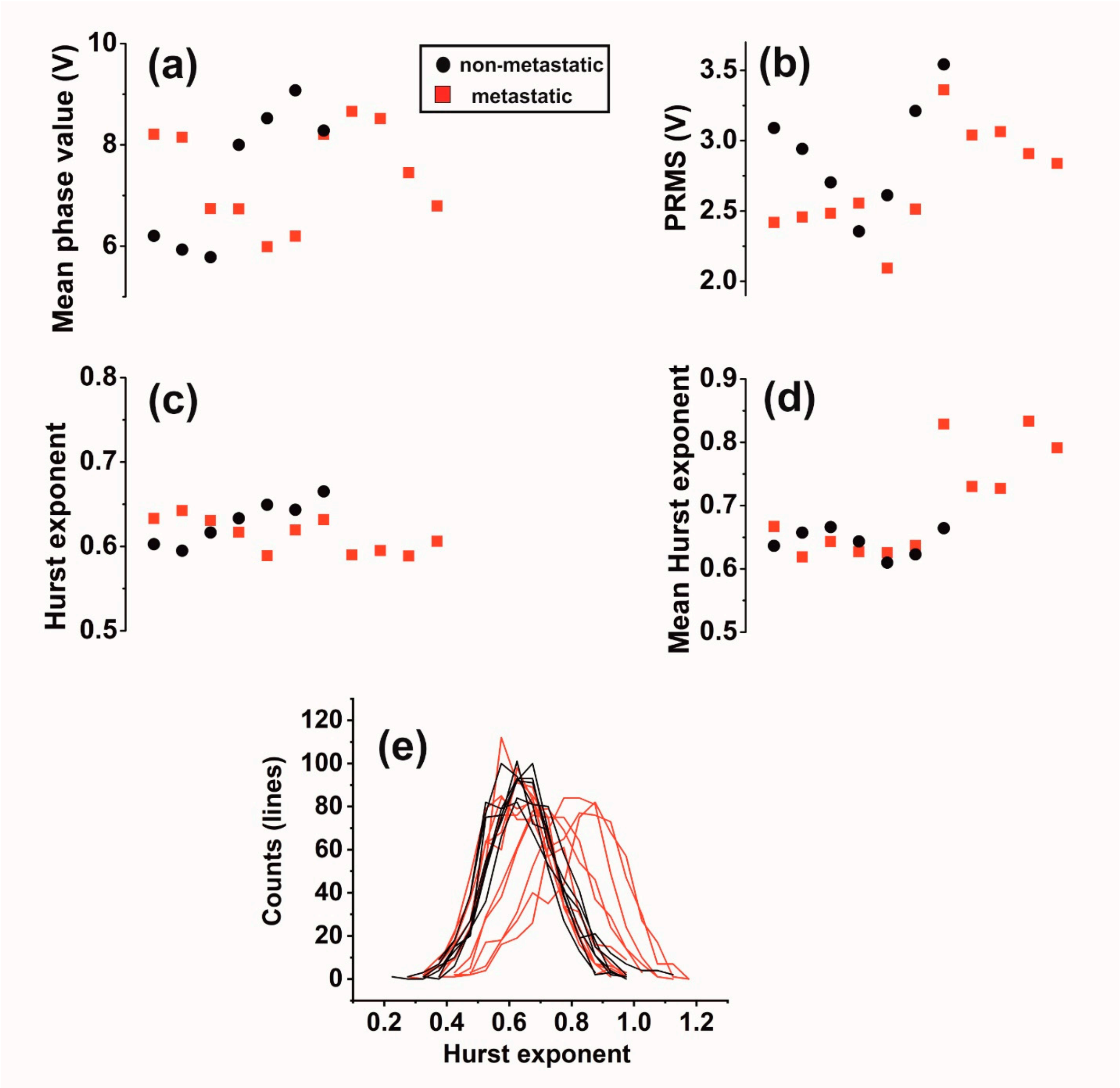

Figure S6. Surface statistical phase spectra of CRC metastatic (red squares) and non-metastatic (black circles) tissue AFM images. **(a)** Mean phase value. **(b)** Phase RMS roughness. **(c)** Hurst exponent. **(d)** Mean Hurst exponent. **(e)** Hurst exponent distribution. The differentiation between metastatic and non-metastatic sections is unclear.

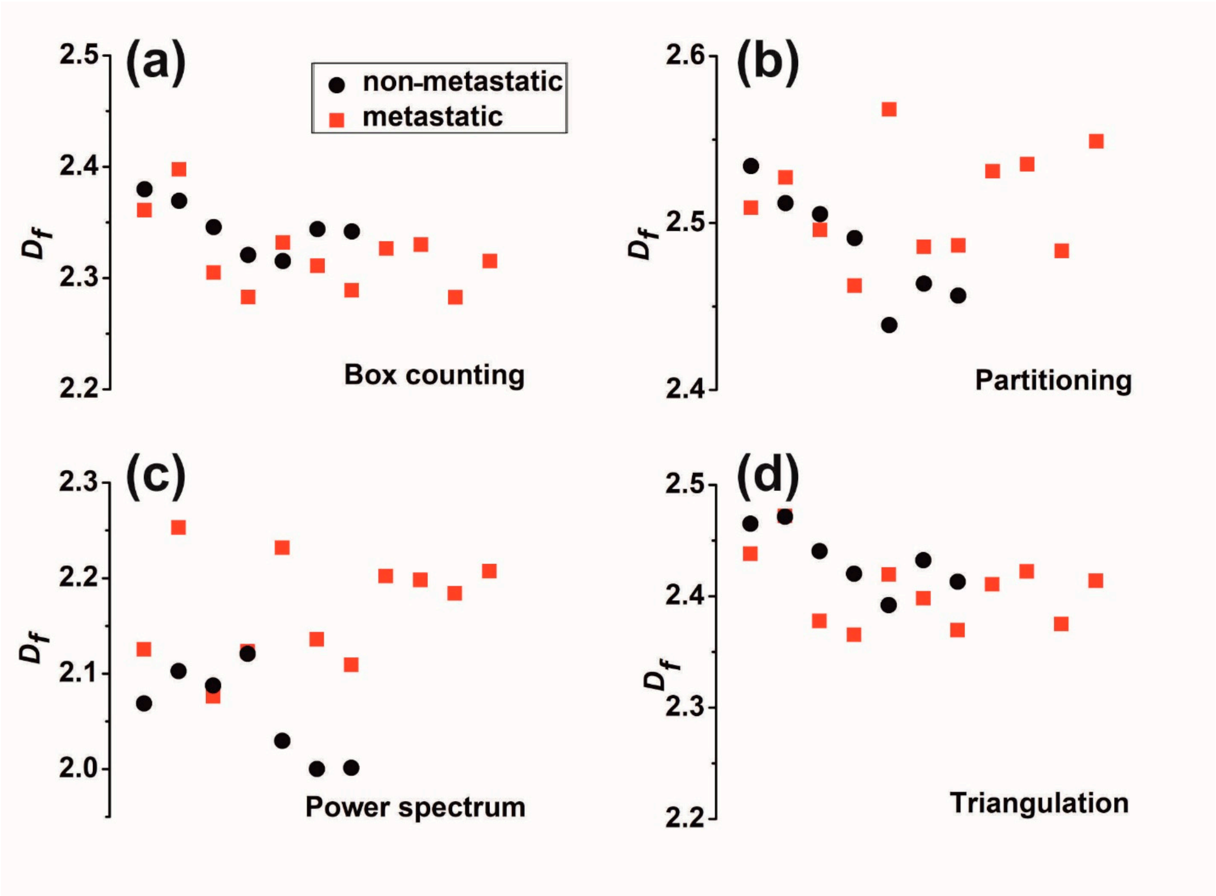

Figure S7. Fractal dimension D_f of images of CRC metastatic (red squares) and non-metastatic (black circles) tissue AFM images were calculated with four different methods. **(a)** Box counting. **(b)** Partitioning. **(c)** Power spectra. One M and one NM point almost coincide **(d)** Triangulation. The differentiation between metastatic and non-metastatic sections is unclear. One M and one NM point almost coincide

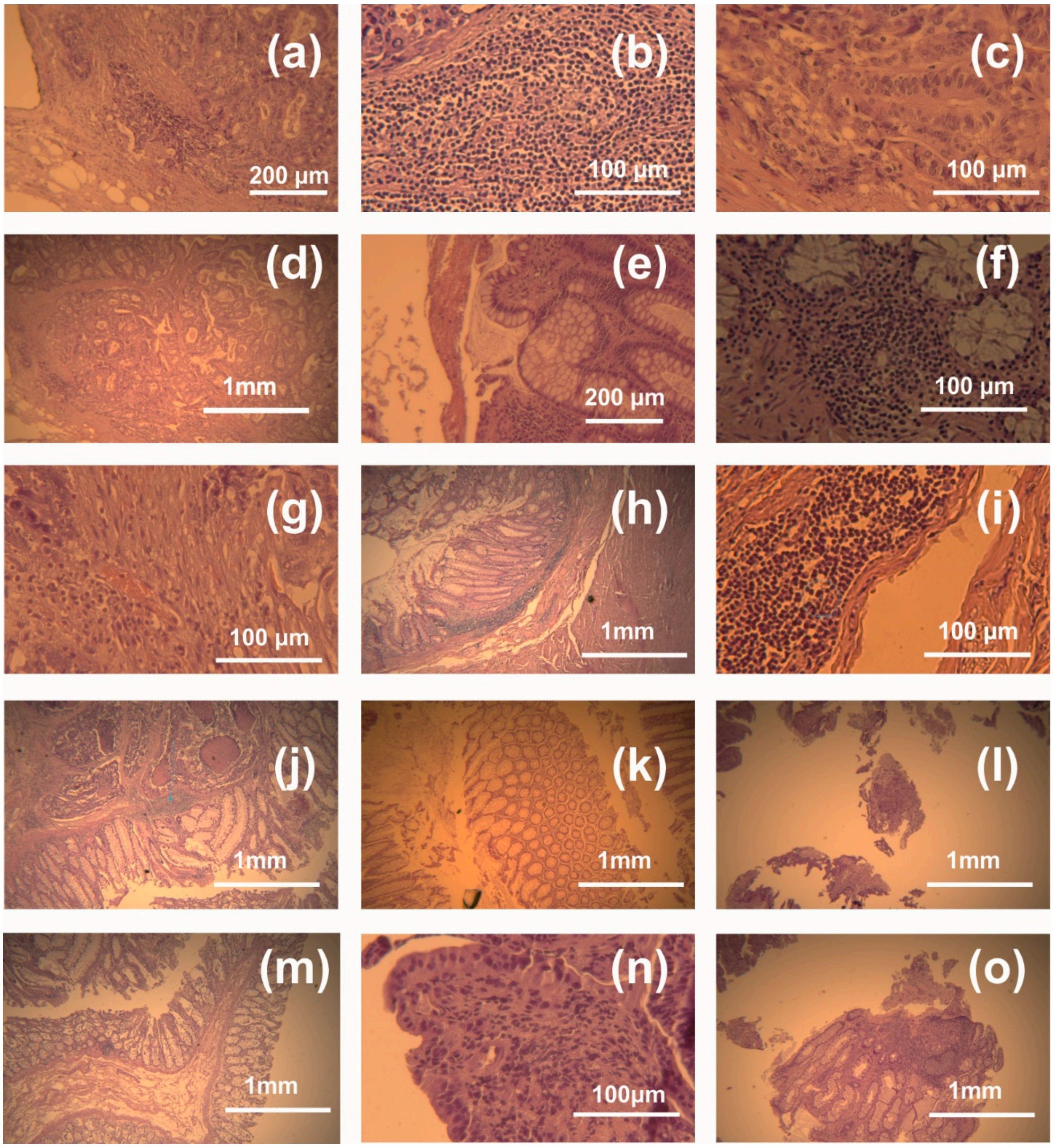

Figure S8. Optical images of metastatic and non-metastatic tissue sections captured with 4x lens (d, h, j, k, l, m, o), 10x (a, e), 40x (b, c, f, g, i, n) magnification.

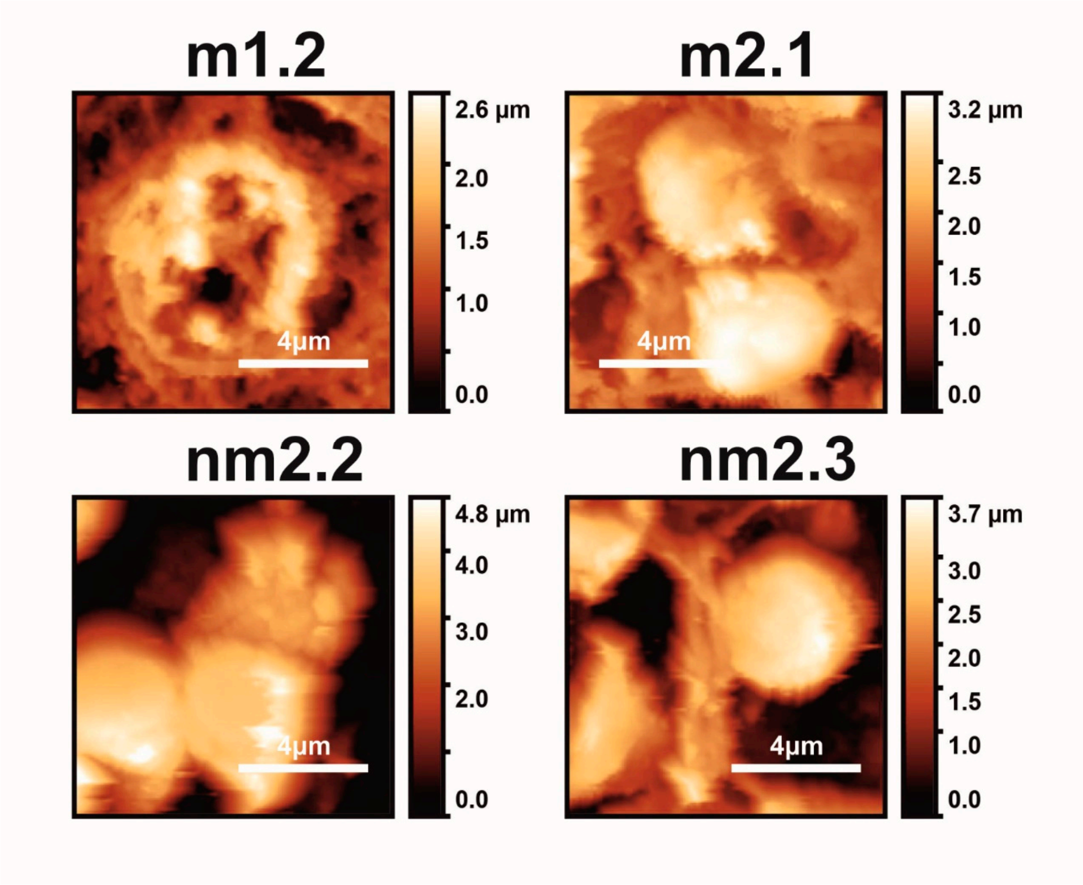

Figure S9. Typical zoomed scanned areas 10 µm x 10 µm of AFM images of Fig.1 of metastatic (m1.2&m2.1) and non-metastatic (nm2.2&nm2.3) human CRC histological sections. The first and second numbers refer to the patient and sample, respectively (m and nm belong to different patients). The color bar in the vertical y-axis represents the z-heights of the image area. The visual differentiation between the metastatic and non-metastatic tissues in AFM images is unclear and is revealed only via image processing (variograms and theta statistics).

S6. Two-sample t-test analysis

The independent two-sample t-test analysis inspects whether or not the means of two independent samples are equal or whether they differ by a pre-defined value and creates a confidence interval for the difference of the sample means. A two-sample t-test analysis was performed, taking the 11 variogram sills of metastatic (M) and 7 variogram sills of non-metastatic (NM) tissues as independent samples. The null hypothesis is that the variogram mean sill values of M and NM samples are equal. Calculations of the *t*-test statistic and p-value determine whether or not to reject the null hypothesis. A smaller p-value means that there is strong evidence in favour of the alternative hypothesis (rejection). Considering that the proposed variogram sill threshold for M or NM characterization is the median value between mean values of M/NM samples variogram sills, the confidence interval for the difference provided by t-test is the confidence interval of the threshold value.

The null hypothesis takes the form $\mu_{NM.} - \mu_M = 0$, while the alternative hypothesis is $\mu_{NM.} - \mu_M \neq 0$. A two-tailed t-test with non-equal variances was performed. An approximate test statistic, t is used:

$$t = \frac{\mu_{NM} - \mu_M}{\sqrt{\frac{s_{NM}^2}{N_{NM}} + \frac{s_M^2}{N_M}}}$$

where μ and s^2 are the means and the variances of the NM and M populations, respectively. A t-distribution with v degrees of freedom is used to approximate the distribution of t where

$$v = \frac{\left(\frac{s_{NM}^2}{N_{NM}} + \frac{s_M^2}{N_M}\right)^2}{\frac{\left(\frac{s_{NM}^2}{N_{NM}}\right)}{N_{NM} - 1} + \frac{\left(\frac{s_M^2}{N_M}\right)}{N_M - 1}}$$

Then the t value is compared to the critical value, and the null hypothesis is rejected if $|t| > t_{\sigma/2}$ where $t_{\sigma/2}$ is the critical value of the t-distribution with v degrees of freedom and σ significance level. The p-value was also compared with σ significance level, 0.05 in our case. The upper and lower $(1 - \sigma) \times 100\%$ confident limits for the mean difference $\mu_{NM} - \mu_M$ are calculated as:

$$\left[(\mu_{NM} - \mu_M) - t_{\frac{\sigma}{2}}\sqrt{\frac{s_{NM}^2}{N_{NM}} + \frac{s_M^2}{N_M}} \, , (\mu_{NM} - \mu_M) + t_{\sigma/2}\sqrt{\frac{s_{NM}^2}{N_{NM}} + \frac{s_M^2}{N_M}} \right]$$

Data for the t-test analysis were taken from Supplementary Table S1, and the t-test results, the corresponding p-values and the threshold confidence intervals are shown in Supplementary Table S2, and Supplementary Table S3.

Resolution	512 px × 512 px						256 px × 256 px						128 px × 128 px					
Sigma [px]	σ=10 px		σ=5 px		σ=2.5 px		σ=10 px		σ=5 px		σ=2.5 px		σ=10 px		σ=5 px		σ=2.5 px	
Sigma [μm]	σ=1 μm		σ=0.5 μm		σ=0.24 μm		σ=2 μm		σ=1 μm		σ=0.5 μm		σ=3.9 μm		σ=2 μm		σ=1 μm	
Sample	Sill (μm)	Range (px)	Sill (μm)	Range (px)	Sill (μm)	Range (px)	Sill (μm)	Range (px)	Sill (μm)	Range (px)	Sill (μm)	Range (px)	Sill (μm)	Range (px)	Sill (μm)	Range (px)	Sill (μm)	Range (px)
m1.1	0.467	21	0.274	12	0.149	7	0.699	16	0.469	11	0.277	6	0.852	12	0.699	8	0.472	6
m1.2	0.395	18	0.254	11	0.152	6	0.533	17	0.395	9	0.256	6	0.680	15	0.534	9	0.397	5
m2.1	0.515	23	0.284	13	0.150	8	0.854	19	0.519	12	0.287	7	1.136	13	0.858	10	0.526	6
m2.2	0.506	23	0.279	13	0.147	8	0.843	19	0.509	12	0.283	7	1.113	13	0.846	10	0.515	6
m3.1	0.410	20	0.245	12	0.135	7	0.603	17	0.412	10	0.248	6	0.765	15	0.603	9	0.414	5
m3.2	0.422	20	0.252	12	0.137	7	0.616	17	0.424	10	0.255	6	0.777	16	0.616	9	0.426	5
m3.3	0.349	19	0.219	11	0.124	7	0.481	15	0.350	10	0.221	6	0.581	15	0.481	8	0.352	5
m3.4	0.446	22	0.258	12	0.139	8	0.683	17	0.449	11	0.261	6	0.902	15	0.685	9	0.453	6
m3.5	0.465	22	0.263	12	0.140	8	0.719	17	0.467	11	0.266	6	0.949	16	0.719	9	0.471	6
m3.6	0.443	21	0.254	12	0.133	9	0.701	20	0.445	10	0.257	6	0.964	15	0.704	10	0.450	5
m3.7	0.441	20	0.262	12	0.142	7	0.646	17	0.443	10	0.265	6	0.806	13	0.646	8	0.446	5
**Mean**	**0.442**	**20.8**	**0.258**	**12.0**	**0.140**	**7.5**	**0.671**	**17.4**	**0.444**	**10.5**	**0.261**	**6.2**	**0.866**	**14.4**	**0.672**	**9.0**	**0.447**	**5.5**
nm1.1	0.692	20	0.381	13	0.191	9	1.061	17	0.696	10	0.386	7	1.275	11	1.063	9	0.701	5
nm1.2	0.697	21	0.377	14	0.188	10	1.100	17	0.701	11	0.383	7	1.356	12	1.104	9	0.708	6
nm1.3	0.757	21	0.403	14	0.199	13	1.165	16	0.761	10	0.409	7	1.383	11	1.167	8	0.767	5
nm2.1	0.633	24	0.339	14	0.180	8	1.123	20	0.637	12	0.343	7	1.511	12	1.128	10	0.645	7
nm2.2	0.670	22	0.362	14	0.188	8	1.165	21	0.674	12	0.367	7	1.570	12	1.169	11	0.681	6
nm2.3	0.651	22	0.350	13	0.182	11	1.118	19	0.656	12	0.356	7	1.490	13	1.120	10	0.662	6
nm2.4	0.482	20	0.280	13	0.150	8	0.722	17	0.484	10	0.283	6	0.925	14	0.723	8	0.486	5
Mean	0.655	21.4	0.356	13.6	0.183	9.6	1.065	18.1	0.658	11.0	0.361	6.9	1.359	12.1	1.068	9.3	0.664	5.7
**Mean (-nm2.4)**	**0.683**	**21.7**	**0.369**	**13.7**	**0.188**	**9.8**	**1.122**	**18.3**	**0.687**	**11.2**	**0.374**	**7.0**	**1.431**	**11.8**	**1.125**	**9.5**	**0.694**	**5.8**
Threshold	0.548		0.307		0.162		0.868		0.551		0.311		1.112		0.870		0.556	
**Threshold (-nm2.4)**	**0.563**		**0.314**		**0.164**		**0.896**		**0.566**		**0.318**		**1.148**		**0.899**		**0.571**	

Supplementary Table S1. Variogram parameters (sill, range), mean value and threshold, the arithmetic mean of sill values of metastatic (M) and non-metastatic (NM) tissues, with and without the nm2.4 sample (red colour), for different AFM image resolution and standard deviations σ in px and μm.

Resolution (px x px)	σ (px)	Tissue	Mean (µm)	StDev (µm)	SE Mean (µm)	D (µm)	StDev of D (µm)	95% CI for D (µm)	Threshold ± (95% CI for D) (µm)	NM ≠ M: p-value
**512 x 512**	**10**	**NM**	0.683	0.043	0.018	0.242	0.046	0.050	**0.563±0.050**	3.14E-07
		**M**	0.442	0.048	0.014					
	**5**	**NM**	0.369	0.023	0.009	0.110	0.020	0.025	**0.314±0.025**	5.60E-06
		**M**	0.258	0.018	0.005					
	**2.5**	**NM**	0.188	0.007	0.003	0.048	0.008	0.008	**0.164±0.008**	2.53E-08
		**M**	0.140	0.008	0.003					
**256 x 256**	**10**	**NM**	1.122	0.040	0.016	0.451	0.096	0.082	**0.896±0.082**	1.49E-08
		**M**	0.671	0.114	0.034					
	**5**	**NM**	0.687	0.043	0.018	0.243	0.047	0.050	**0.566±0.050**	2.62E-07
		**M**	0.444	0.049	0.015					
	**2.5**	**NM**	0.374	0.024	0.010	0.112	0.020	0.026	**0.318±0.026**	5.64E-06
		**M**	0.261	0.018	0.006					
**128 x 128**	**10**	**NM**	1.431	0.111	0.045	0.565	0.153	0.147	**1.148±0.147**	8.08E-07
		**M**	0.866	0.170	0.051					
	**5**	**NM**	1.125	0.040	0.016	0.453	0.097	0.083	**0.899±0.083**	1.65E-08
		**M**	0.672	0.115	0.035					
	**2.5**	**NM**	0.694	0.043	0.017	0.246	0.048	0.051	**0.571±0.051**	1.84E-07
		**M**	0.447	0.050	0.015					

Supplementary Table S2. Statistical parameters of sill values of non-metastatic (NM) and metastatic (M) tissues for different Gaussian Filter Sigma σ (px). Mean sill values, Standard Deviation (StDev), Standard Error of Mean (SE Mean), the difference of M and NM mean values (D), Standard Deviation of Difference (StDev of D), 95% Confidence Interval for the difference (95% CI for D), Threshold ± 95%CI for D, p-value statistic parameter for the hypothesis NM ≠ M.

	Tissue	Mean	StDev	SE Mean	D	StDev of D	95% CI for D	NM≠M : p-value
**Theta distribution skewness**	**NM**	-0.138	0.218	0.082	0.566	0.191	0.217	0.000128
	**M**	0.428	0.174	0.052				
**Theta distribution kurtosis**	**NM**	1.857	0.178	0.067	0.339	0.190	0.194	0.00210
	**M**	2.195	0.197	0.059				
**Average *z*-height (nm)**	**NM**	2061.7	486.2	146.6	102.6	416.1	376.3	0.569
	**M**	2164.3	260.2	98.3				
**RMS roughness (nm)**	**NM**	689.6	127.4	38.4	306.0	130.5	139.6	0.000422
	**M**	995.6	135.7	51.3				
**Mean Hurst exponent of 512 lines (topo).**	**NM**	0.860	0.040	0.013	-0.040	0.037	0.037	0.0369
	**M**	0.820	0.031	0.012				
**Hurst exponent of 512 l lines (topo)**	**NM**	0.603	0.040	0.013	0.013	0.039	0.041	0.509
	**M**	0.616	0.038	0.014				
**Phase average value (*V*)**	**NM**	7.422	0.970	0.292	-0.023	1.141	1.353	0.969
	**M**	7.399	1.380	0.522				
**Phase "RMS roughness" (*V*)**	**NM**	2.703	0.368	0.111	0.219	0.381	0.409	0.265
	**M**	2.922	0.401	0.152				
**Mean Hurst exponent of 512 lines (Phase)**	**NM**	0.703	0.084	0.025	-0.060	0.067	0.058	0.0437
	**M**	0.643	0.021	0.008				
**Hurst exponent of 512 lines as one line (Phase)**	**NM**	0.613	0.020	0.006	0.016	0.022	0.026	0.182
	**M**	0.629	0.026	0.010				
**FD Box Counting**	**NM**	2.321	0.035	0.010	0.024	0.031	0.029	0.0976
	**M**	2.345	0.023	0.009				
**FD Partitioning**	**NM**	2.512	0.032	0.010	-0.026	0.033	0.035	0.131
	**M**	2.486	0.034	0.013				
**FD Triangulation**	**NM**	2.406	0.033	0.010	0.028	0.031	0.031	0.0762
	**M**	2.434	0.028	0.011				
**FD Power Spectrum**	**NM**	2.168	0.056	0.017	-0.109	0.054	0.054	0.000633
	**M**	2.059	0.049	0.018				

Supplementary Table S3. Statistical parameters of theta distribution skewness (Figure 6b) and kurtosis (Figure 6c), average *z*-height (nm) (Figure S5a), RMS roughness (nm) (Figure S5b), Mean Hurst exponent of 512 lines (topography) (Figure S5c), Hurst exponent of 512 lines as one line (topography) (Figure S5d), average phase value (*V*) (Figure S6a), Phase "RMS roughness" (*V*) (Figure S6b), Mean Hurst exponent of 512 lines (phase) (Figure S6c), Hurst exponent of 512 lines as one line (phase) (Figure S6d), Fractal Dimension Box Counting (Figure S7a), Partitioning (Figure S7b), Triangulation (Figure S7c) and Power Spectrum (Figure S7d) methods, of non-metastatic (NM) and metastatic (M) tissues. Mean values, standard deviation (StDev), standard error of the mean (SE Mean), the difference of M and NM mean values (D), standard deviation of difference (StDev of D), 95% Confidence Interval for the difference (95%CI for D), p-Value statistic parameter for the hypothesis NM ≠ M.

References

46. Velentzas, A.D.; Velentzas, P.D.; Katarachia, S.A.; Anagnostopoulos, A.K.; Sagioglou, N.E.; Thanou, E.V.; Tsioka, M.M.; Mpakou, V.E.; Kollia, Z.; Gavriil, V.E.; et al. The indispensable contribution of s38 protein to ovarian-eggshell morphogenesis in Drosophila melanogaster. *Sci. Rep.* **2018**, *8*, 16103. https://doi.org/10.1038/s41598-018-34532-2.
60. Nečas, D.; Klapetek, P. Gwyddion: an open-source software for SPM data analysis. *Open Phys.* **2012**, *10*, 181–188. https://doi.org/10.2478/s11534-011-0096-2.
61. Hurst, H.E. Long-Term Storage Capacity of Reservoirs. *Trans. Am. Soc. Civ. Eng.* **1951**, *116*, 770–808, https://doi.org/10.1061/taceat.0006518.
62. HURST: MATLAB function to compute the Hurst exponent using R/S Analysis. Available online: https://ideas.repec.org/c/wuu/hscode/m11003.html
